# FGF signalling plays similar roles in development and regeneration of the skeleton in the brittle star *Amphiura filiformis*

**DOI:** 10.1242/dev.180760

**Published:** 2021-05-27

**Authors:** Anna Czarkwiani, David V. Dylus, Luisana Carballo, Paola Oliveri

**Affiliations:** 1 Department of Genetics, Evolution and Environment, University College London, London WC1E 6BT, UK; 2 Centre for Mathematics, Physics and Engineering in the Life Sciences and Experimental Biology, University College London, London WC1E 6BT, UK; 3 Centre for Life's Origin and Evolution (CLOE), University College London, London WC1E 6BT, UK

**Keywords:** Echinoderm, Biomineralization, Regulatory networks, Signalling, Vegf

## Abstract

Regeneration as an adult developmental process is in many aspects similar to embryonic development. Although many studies point out similarities and differences, no large-scale, direct and functional comparative analyses between development and regeneration of a specific cell type or structure in one animal exist. Here, we use the brittle star *Amphiura filiformis* to characterise the role of the FGF signalling pathway during skeletal development in embryos and arm regeneration. In both processes, we find ligands expressed in ectodermal cells that flank underlying skeletal mesenchymal cells, which express the receptors. Perturbation of FGF signalling showed inhibited skeleton formation in both embryogenesis and regeneration, without affecting other key developmental processes. Differential transcriptome analysis finds mostly differentiation genes rather than transcription factors to be downregulated in both contexts. Moreover, comparative gene analysis allowed us to discover brittle star-specific differentiation genes. In conclusion, our results show that the FGF pathway is crucial for skeletogenesis in the brittle star, as in other deuterostomes, and provide evidence for the re-deployment of a developmental gene regulatory module during regeneration.

## Abstract (precis)

**Summary:** Cellular and molecular analyses of FGF signalling perturbation during embryonic development and arm regeneration of the brittle *Amphiura filiformis* identify a conserved role in the formation of the skeleton in both processes.

## INTRODUCTION

A tempting theory for the evolutionary origins of tissue regeneration suggests it was selected as a secondary by-product of development, thus sharing many similarities with embryogenesis (Brockes and Kumar, 2008; Morgan, 1901). In fact, following the unique processes of regeneration (such as wound healing and dedifferentiation), cell specification and differentiation must occur just as they do during embryonic development. Studies have shown that gene expression during development and regeneration can be conserved. For example, in newt the sonic hedgehog gene recapitulates its role in developing limb buds during adult regeneration (Imokawa and Yoshizato, 1997), as it does during elbow joint regeneration in developing chick embryos (Özpolat et al., 2012). Meis genes under control of the retinoic acid signalling pathway are also involved in salamander limb regeneration, similar to their role during embryonic limb development (Mercader et al., 2005). In planarians, many of the components of the genetic network underlying eye development in other species (e.g. *otx*, *six*, *opsin*) have been shown to be expressed and functionally required during adult eye regeneration, although others (i.e. *pax6*) play no role in this context, underlying some important differences (Pineda et al., 2002; Saló et al., 2002). Unravelling the function of signalling pathways and transcription factors (TFs) in development and regeneration can thus shed light on whether adult organisms with the capability of regeneration re-use developmental gene regulatory networks (GRNs). However, few studies exist, and these mostly compare the expression of a single gene between development and regeneration in the same organism. With new transcriptomic databases (e.g. Iberian ribbed newt, Matsunami et al., 2019; sea anemone, Warner et al., 2018) comparative analysis has shown that embryonic GRNs are partially re-used during adult sea anemone whole-body regeneration. Consistent with the idea that the initiation of regeneration is very different from embryonic development, several genes have been identified that are unique to regeneration (Warner et al., 2019 preprint).

Comparing the role of signalling pathways in embryogenesis and regeneration provides a compelling strategy to understand the extent of similarities between GRNs driving these two developmental processes. A good example of this is the fibroblast growth factor (FGF) signalling pathway, implicated in a wide range of biological processes such as cell migration, differentiation and proliferation, during development, wound healing and regeneration (Thisse and Thisse, 2005; Dorey and Amaya, 2010). Regeneration in hydra, zebrafish, *Xenopus* and salamanders relies on the expression of FGF genes, and applying FGF receptor (FGFR) inhibitors results in regenerative defects (Poss et al., 2000; Lee et al., 2005; Lin and Slack, 2008; Makanae et al., 2014; Shibata et al., 2016; Turwankar and Ghaskadbi, 2019). The FGF signalling pathway also plays important roles in development and regeneration of the vertebrate skeleton. Mutations in both ligands and receptors were found to cause a variety of congenital disorders including craniosyntoses, chondrodysplasia (Cunningham et al., 2007; Marie et al., 2005; Roscioli et al., 2000) and multiple types of gross skeletal abnormalities in mouse models and humans (Teven et al., 2014). Similarly, multiple FGFs and FGFRs are expressed during fracture healing and bone regeneration (Schmid et al., 2009). Importantly, the precise roles and effects of FGF inhibition during postembryonic morphogenesis are not well understood (Du et al., 2012).

The role of FGF signalling in skeletogenesis also extends to echinoderms, which are an excellent experimental system for studying the GRNs underlying development (Oliveri et al., 2008; Davidson et al., 2002; Peter and Davidson, 2010; Annunziata and Arnone, 2014). FGF signalling is necessary for guiding skeletogenic mesenchymal cell migration and formation of the embryonic skeleton in the sea urchin *Paracentrotus lividus* (Röttinger et al., 2008). Interestingly, in a different species, *Lytechinus variegatus*, FGF inhibition using *fgf9/16/20* (*fgf*) morpholino (also called *fgfa*) produces a much milder phenotype in comparison with *P. lividus* (Röttinger et al., 2008), whereby the mesenchymal cells migrate normally and the embryos form shortened skeletal rods (Adomako-Ankomah and Ettensohn, 2013). In addition to FGF, the VEGF signalling pathway is also involved in skeletogenesis in both species. Perturbation of the *vegf3* (*vegf3l*) ligand in the sea urchin interferes with both correct skeletogenic cell migration and skeletal rod formation (Adomako-Ankomah and Ettensohn, 2013; Duloquin et al., 2007; Erkenbrack and Petsios, 2017). It appears to be clear that both of these pathways have essential, often interconnected and non-redundant, roles in skeletogenesis in the sea urchin embryo. However, whether these pathways regulate different downstream effector genes, and whether their role is conserved during adult skeletogenesis in echinoderms remain unknown.

Recently, several studies have established the brittle star *Amphiura filiformis* (*Afi*) as an experimental system for skeleton formation in both embryonic development (Dylus et al., 2016, 2018) and adult regeneration (Czarkwiani et al., 2013, 2016). Characterization of these processes showed that the skeletogenic cells of both the adult and embryo are mesenchymal and express an array of skeletogenic specification TFs such as *alx1*, a gene that has a conserved role in skeleton development in echinoderms (Ettensohn et al., 2003) and vertebrates (Brouwer et al., 2003; ten Berge et al., 1998). Adult skeletogenic cells also express downstream embryonic skeletal differentiation genes, including *c-lectin* (*clec19a*), *p58b*, *p19* (*P19L*) and *αcoll* (*col2a1*) (Dylus et al., 2016; Czarkwiani et al., 2013, 2016). Moreover, transcriptomic data for both the embryonic stages (Dylus et al., 2018; Delroisse et al., 2015) and the adult regenerating and non-regenerating arms (Purushothaman et al., 2014; Burns et al., 2011) are now available. With this wealth of information on regeneration and early development of the skeleton, this species can be used to directly compare the role of FGF signalling in both processes within the same animal at different life stages.

In this study, we carry out a large-scale, side-by-side comparison of the development of the skeleton during embryogenesis and adult regeneration in the context of FGF signalling. We first characterize the expression of FGF signalling components during morphologically comparable stages of the development of the skeleton in both processes. We then use an FGF signalling inhibitor (SU5402) in embryos and adult *A. filiformis* to determine the effect of disrupting this pathway. We find that perturbation of FGF signalling in brittle stars results in failure to form skeletal spicules in both the embryos and in the regenerating arms. Using an unbiased transcriptome approach comparing control and treated embryos, we find several brittle star-specific skeletogenic genes. Moreover, many of these are affected similarly in embryos and in adult regenerating arms, suggesting a conservation of pathway components and network connections between these two processes. Ultimately, our study provides the first direct evidence for an analogous role of FGF signalling in skeletogenesis between embryonic development and adult regeneration in the same species working downstream from the specification tier of the skeletogenic GRN.

## RESULTS

### Evolutionary relationships of FGF and VEGF signalling components in echinoderms

Both Fgf and Vegf signalling pathways are required in the development of the sea urchin larval skeleton (Czarkwiani et al., 2013, 2016; Ettensohn et al., 2003; Brouwer et al., 2003). To characterize signalling genes in these two pathways in *A. filiformis*, we first surveyed an embryonic transcriptome encompassing the entirety of development (from cleavage stage to pluteus larvae) (Dylus et al., 2018) and transcriptomes from adult regenerating and non-regenerating arms (Purushothaman et al., 2014) of *A. filiformis* for potential homologs. To do this, we combined a BLAST search using selected candidates (e-value 1e-6) from the sea urchin database (Cameron et al., 2009) with a hidden Markov model search against PFAM domains of Fgf and Vegf ligands and receptors (Finn et al., 2016). Using this strategy, we found two potential Fgf ligands, three Fgf receptors, two Vegf ligands and one Vegf receptor in *A. filiformis* (Table S1).

To better understand the evolutionary relationships of our *A. filiformis* genes relative to echinoderm and chordate signalling systems, we used a collection of sequences of Fgf and Vegf ligands and receptors for 41 species spanning all major clades of echinoderms, chordates (e.g. mouse, rat etc.) and non-deuterostome outgroup species such as the pacific oyster (*Crassostrea gigas*). For each of the four datasets we computed the orthologous relationships using the Orthologous Matrix Algorithm (OMA) (Altenhoff et al., 2019) and extracted five groups containing our genes (see Materials and Methods for details). For each of these groups we then computed maximum likelihood phylogenetic trees using amino acid sequences. We observed that two Fgf ligands were placed in an echinoderm group sharing a common ancestor with their respectively independently duplicated genes in chordates (Figs S1, S2). The evolutionary relationship with the well-studied sea urchin orthologous sequences is well supported. For example, *Afi-fgf9/16/20* shares a highly supported common ancestor with the *Strongylocentrotus purpuratus* *Spu-fgf9/16/20* (*fgf*) gene (Fig. S1), whereas the relation with chordates and hemichordates has low support values, despite the OMA run identifying a clear gene group with the vertebrate FGF9, FGF16 and FGF20. A similar result is obtained for the ligand gene *Afi-fgf8/17/18* (*fgfl*), with a better support to the chordate genes (Fig. S2).

Different evolutionary relationships were revealed for the Fgf receptors. Three *A. filiformis* sequences were identified in the FGFR OMA group, which includes chordate FGF receptors. Afi-Fgfr1, Afi-Fgfr2 and Afi-Tk9 were all in a group with other echinoderm FGF receptors, which includes the sea urchin Sp-Fgfr (*fgfr3*; also known as Fgfr1; Lapraz et al., 2006), Sp-Fgfr2 (*fgfr2l*; also known as Fn3_Ig_29; Lapraz et al., 2006) and Sp-Tk9, respectively. All echinoderm FGF receptors had a weak relation to the group of chordate FGFR1, FGFR2 and FGFR4 receptors (Fig. S3). Our analysis suggests an evolutionary scenario in which both chordates and echinoderms independently duplicated these genes from a single common ancestor. FGF receptors are membrane proteins, with an extracellular domain consisting of three immunoglobulin-like subdomains (Ig), a transmembrane (TM) domain, and an intracellular region encompassing a tyrosine kinase domain (PTK) (Fig. S3) (Sarabipour and Hristova, 2016). A protein conserved domain analysis conducted on the three potential *A. filiformis* FGF receptors showed that only Afi-Fgfr1 and Afi-Fgfr2 are equipped with all the structural domains to work as FGF receptors; Afi-TK9 is not. Therefore, only two FGF receptors were identified in *A. filiformis*, consistent with what has already been reported in other echinoderms (Lapraz et al., 2006; Fan and Su, 2015). For the Vegf ligands, we also observed independent duplication events in chordates (VEGFA and VEGFB) as well as echinoderms (Vegf2 and Vegf3) (Fig. S4). Both *A. filiformis* Vegf ligands share a highly supported common ancestor with their annotated genes in sea urchin. The only VEGF receptor of echinoderms forms a sister group to three VEGF receptor genes in chordates (Fig. S5). Here, however, we specifically focused on genes with clear orthology between the sea urchin and brittle star, as the sea urchin has a well-annotated genome. Ultimately, this analysis highlighted the difficulties of drawing clear orthology among distantly related species when clade-specific gene duplication and losses occurred, but importantly allows us to bring our results into a broader evolutionary context when comparing across different species.

### FGF signalling genes are expressed during both embryonic development and adult arm regeneration

**Fig. 1.**
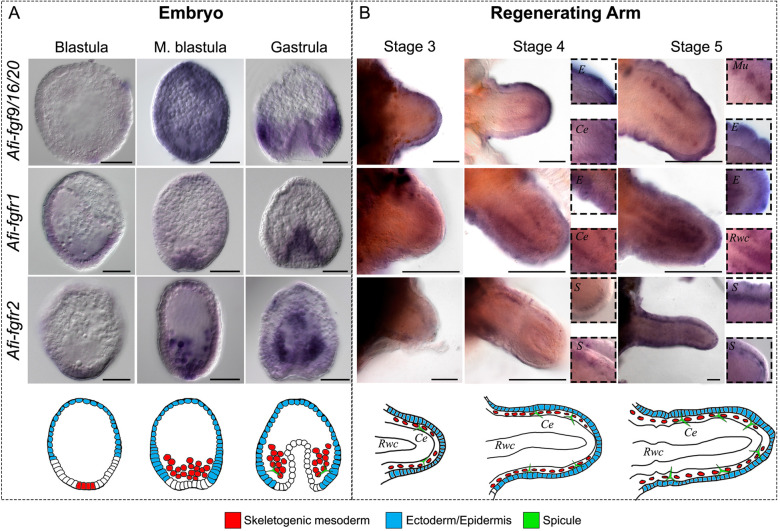
**Expression of FGF signalling components in embryos and early regenerating arm stages of *A. filiformis*.** (A) Top: WMISH on embryos at blastula, mesenchyme blastula and gastrula stages of development showing expression of *Afi-fgf9/16/20*, *Afi-fgfr1* and *Afi-fgfr2*. Bottom: schematic of major relevant cellular domains in corresponding stage embryos. (B) Top: WMISH on regenerates at stages 3, 4 and 5 showing the expression of *Afi-fgf9/16/20*, *Afi-fgfr1* and *Afi-fgfr2.* Insets show detail of expression patterns. Bottom: Schematic of major relevant cellular domains at corresponding stages. Ce, coelomic epithelium; E, epidermis; Mu, metameric units; Rwc, radial water canal; S, skeletogenic cells in dermal layer. Images from the aboral view. Scale bars: 50 μm (A); 100 μm (B).

To better understand the role of FGF signalling genes in the context of brittle star skeletogenesis, we first analysed the expression of ligands and receptors during embryogenesis and adult regeneration using *in situ* hybridization (ISH) and NanoString transcript quantification. For this purpose, we selected the most likely corresponding stages between development and regeneration using the established staging system for regenerating arms (Czarkwiani et al., 2016) and the developmental timeline for embryos (Dylus et al., 2016) (Fig. S6A,B); and gene activity (Fig. S7A). Specifically, we focused on developmental stages when the skeletogenic lineage is segregated from other mesodermal cells and specific skeletogenic genes are expressed (blastula and mesenchyme blastula stages, Fig. 1A, Fig. S6A; stage 3 during arm regeneration, Fig. 1B, Fig. S6B) and when skeletal spicules appear (gastrula stage, Fig. 1A, Fig. S6A; stage 3-5 during adult arm regeneration, Fig. 1B, Fig. S6B) (Dylus et al., 2016, 2018; Czarkwiani et al., 2013, 2016).

**Fig. 2.**
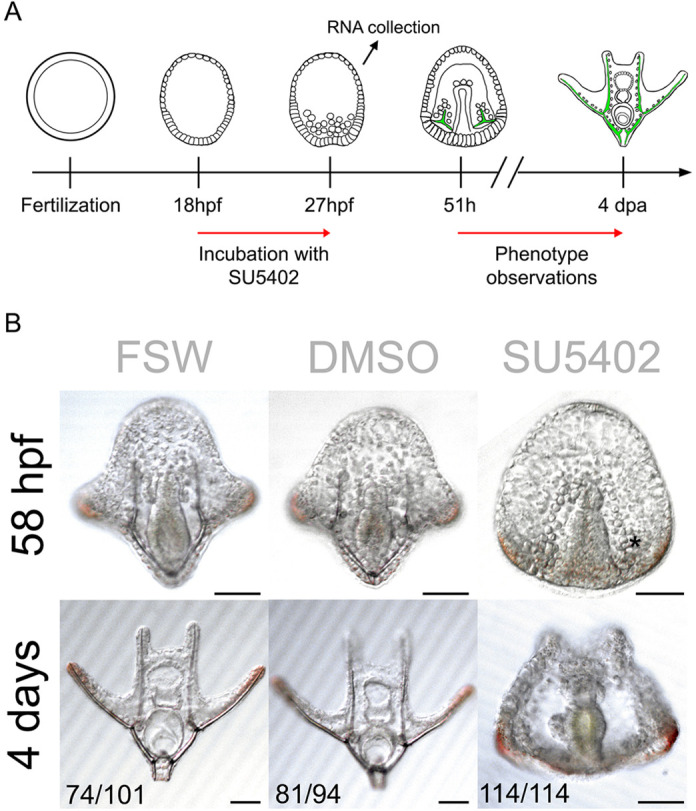
**FGF signalling perturbation using the SU5402 inhibitor in brittle star embryos.** (A) Experimental procedure for SU5402 treatment. (B) Phenotypic analysis of SU5402-treated *A. filiformis* embryos and controls at 58 hpf and 4 days post fertilization shows that perturbation of FGF signalling results in embryos with no skeletal spicules forming. Numbers at the bottom show counts for embryos observed with the represented phenotype/total embryos counted. Scale bars: 50 μm.

In the embryo, the *Afi-fgf9/16/20* ligand was first detectable at mesenchyme blastula stage, between 15 and 18 h post fertilization (hpf) ubiquitously (Fig. 1A; Fig. S7A). It then became confined to a band in the ectodermal domain at the boundary of the endoderm, with higher expression in two domains adjacent to the clusters of mesenchymal cells that will produce the skeleton of the embryo at gastrula stage (Fig. 1A; Fig. S7A). After arm amputation, there was a subtle global upregulation of *Afi-fgf9/16/20* at 48 h post amputation (hpa) followed by a constant level of expression detectable using NanoString (Fig. S7). Whole-mount *in situ* hybridization (WMISH) revealed that *Afi*-*fgf9/16/20* is expressed in the epidermis throughout early regenerative stages (stages 3-5; Fig. 1B). At stage 4 an additional domain adjacent to the radial water canal (RWC) became visible, within the domain of the coelomic epithelium (Ce). This expression was visible in patches of cells in the most proximal part of the stage 5 arm in correspondence to where the newly forming metameric units appeared in the regenerating arm (Fig. 1B). During development, the receptor *Afi-fgfr1* was expressed in the vegetal half of the embryo at blastula stage, and in endoderm and non-skeletogenic mesoderm from mesenchyme blastula stage to gastrula stage (Fig. 1A; Fig. S7A). Upon amputation and after an initial drop in level of expression at 24 hpa, *Afi-fgfr1* levels increased at around stage 3 of regeneration (Fig. S7A) and exhibited a highly dynamic pattern during adult arm regeneration in several territories including the epidermis, radial nerve cord, coelomic epithelium and radial water canal (Fig. 2B; Fig. S6E). Conversely, the receptor *Afi-fgfr2* was first specifically expressed in the skeletogenic mesoderm (SM) cells at mesenchyme blastula stage and expanded to the non-skeletogenic mesoderm at gastrula stage during embryonic development (Fig. 1A; Fig. S7A). During stages 4 and 5 of regeneration it was expressed in the dermal layer, in which the skeleton first appeared during regeneration (Fig. 1B; Fig. S6B). Notably, global expression of *Afi-fgfr2* was relatively low in the NanoString data, which most likely reflects its specific expression only in the small population of skeletogenic cells relative to the whole arm structure (Fig. S7A). Expression of FGF signalling pathway components at late stages of regeneration persisted in similar territories (epidermis for the *fgf9/16/20* ligand and skeletal domains for *fgfr2* receptor; Fig. S8). Other identified components of FGF signalling (*afi-fgf8/17/18* and *afi-tk9*) were not expressed either in cell types or at developmental/regenerative time points relevant to this study (Fig. S7). Interestingly, the expression of *fgf9/16/20* in the ectoderm and the *fgfr2* receptor in mesenchymal cells was comparable with the expression of their orthologues in sea urchin development (Röttinger et al., 2008). With respect to the organization of mesenchymal cells and their proximity to the ligand-expressing ectodermal domain cells, sea urchin and brittle star embryos share a similar topology.

### FGF signalling perturbation with SU5402 inhibits skeleton formation in both embryos and adult regenerating arms

To analyse the role of FGF signalling in skeletogenesis during brittle star embryonic development and adult arm regeneration we applied the SU5402 inhibitor, a small molecule well-known to specifically inhibit the function of FGFRs by competing with ATP for the binding site of the catalytic domain of tyrosine kinase (Mohammadi et al., 1997). This inhibitor has been successfully used to disrupt FGF signalling during both embryogenesis and regeneration in many organisms (Lin and Slack, 2008; Saradamba et al., 2013; Hu and Marcucio, 2012; Eblaghie et al., 2003).

For developmental characterization, we treated brittle star embryos with SU5402, alongside non-treated filtered seawater (FSW) and DMSO (used as the solvent for the drug) controls at 18 hpf preceding SM ingression (Dylus et al., 2016). After an initial test at three different concentrations (5 µM, 10 µM and 20 µM), 10 µM of SU5402 was chosen as the optimal dilution to elicit a consistent and reproducible phenotype without arresting development. The 18 hpf time point was used to avoid interfering with potential early functions of FGF signalling during cleavage stages and to specifically focus on skeletogenesis (as this also corresponds to the temporal onset of *fgfr2* expression in skeletogenic cells, see Fig. 1A and Fig. S7A). At 27 hpf we collected the embryos for RNA-seq and NanoString analysis (Fig. 2A) to assess the early response to signalling inhibition and to avoid secondary effects of FGF perturbation for a prolonged period. At this stage, treated embryos are indistinguishable from controls showing a timely ingression of the primary mesenchymal cells. Subsequently, we scored several embryos at late gastrula and pluteus stages for the formation of spicules. All SU5402-treated embryos failed to develop skeletal spicules (100%, *n*=114), compared with 0.2% DMSO (13.8%, *n*=94) and FSW controls (26.7%, *n*=101) (Fig. 2B). Despite having no visible defects in SM ingression, archenteron invagination or overall survival (58 hpf; Fig. 2B), the perturbed embryos did not develop a skeleton, even at late stages of development (4 days post fertilization; Fig. 2B).

**Fig. 3.**
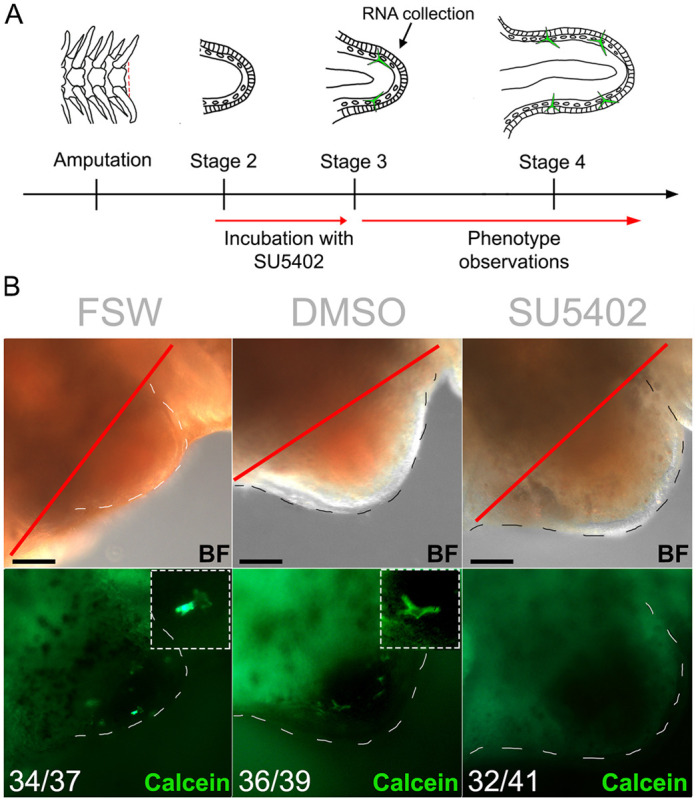
**FGF signalling perturbation using SU5402 in brittle star regenerating arm explants.** (A) Experimental procedure for SU5402 treatment. (B) Phenotypic analysis of SU5402-treated *A. filiformis* regenerates and controls at 24 h post treatment (stage 3) shows that perturbation of FGF signalling inhibits spicule formation. Insets show detail of spicules. Numbers at the bottom show counts for explants observed with the represented phenotype/total explants counted. Red line, amputation plane. Dashed lines, outline of regenerating bud. Scale bars: 50 μm.

**Fig. 4.**
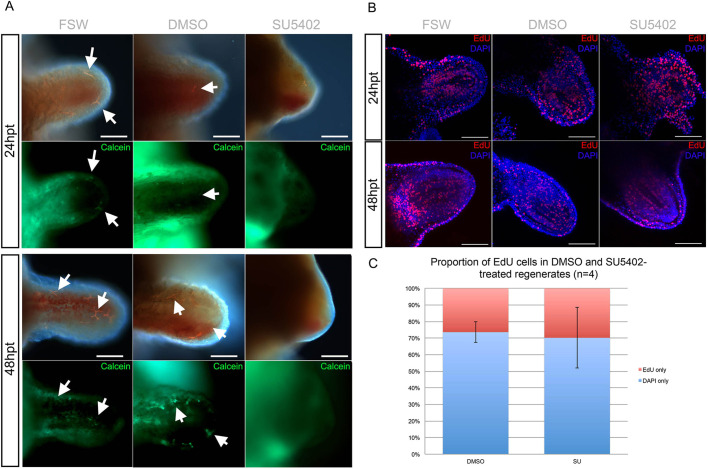
**FGF signalling perturbation interferes with arm regeneration in *A. filiformis* but not by reducing cell proliferation**. (A) Phenotypic analysis of regenerating arm explants in control and SU5402 conditions at 24 h post treatment (hpt) and 48 hpt shows that skeletogenic spicules do not form and the arm ceases to regenerate further. Newly formed skeletal spicules are labelled by calcein in green. Arrows indicate spicules. (B) Confocal images of an EdU cell proliferation assay on control and treated regenerates shows no changes in the proportion of EdU-labelled nuclei in SU5402-treated explants both at 24 hpt and 48 hpt. (C) Quantification of the results in B showing no significant decrease in the proportion of EdU-labelled nuclei relative to all nuclei counted. Error bars show s.e.m. Scale bars: 100 μm.

A similar treatment was performed in regenerating arms to functionally assess the role of FGF signalling during adult skeleton regeneration. We applied the SU5402 inhibitor to amputated arm explants, which can survive separated from the main body for several weeks and continue to regenerate properly (Burns et al., 2012). The explants were incubated in 10 µM SU5402 from stage 2 (before formation of skeletal spicules and the onset of *fgfr2* in dermal cells; Czarkwiani et al., 2016) for 24 h, after which they were scored for phenotype and collected for further analyses (Fig. 3A). FGF signalling perturbation using this method caused inhibition of skeletal spicule formation in the majority of arms (78.1%, *n*=41), as shown by the absence of calcein staining in the dermal layer, compared with 0.1% DMSO controls (7.7%, *n*=39) and non-treated FSW controls (8.1%, *n*=37) (Fig. 3B). All arm explants were alive and mobile after treatment (Movie 1), however only the DMSO and FSW controls continued to regenerate 48 h after treatment (Fig. 4A). As treated explants did not elongate, and to rule out possible toxic side effects, we examined whether the explants retained cell proliferation ability. Interestingly, even though SU5402-treated explants failed to regenerate further (*n*=8; Fig. 4A), we found that cell proliferation was not affected by the inhibition of FGF signalling (*n*=4; Fig. 4B,C). This provided evidence that not all cellular mechanisms have been affected by the treatment, but rather a specific effect has been exerted on regeneration of different tissues, including the skeleton. Importantly, the application of SU5402 led to a reduction of skeleton during both development and regeneration.

### VEGF signalling perturbation with Axitinib mildly inhibits skeleton formation in both embryos and adult regenerating arms of *A. filiformis*

The VEGF signalling pathway plays a pivotal role in sea urchin skeletogenesis, and the expression of its ligand in the ectoderm and of its receptor in mesenchymal cells resembles the expression of components of the FGF pathway (Kipryushina et al., 2013). SU5402 has been shown to have some mild inhibitory effects on this pathway at high concentrations (50µM and above; Sun et al., 1999); therefore to determine to what extent inhibition of the VEGF pathway alone could interfere with skeletogenesis, we characterized the expression of its components (Figs S7B, S9A,B) and inhibited it with a VEGF specific inhibitor, Axitinib, which selectively inhibits VEGF receptors by blocking their cellular autophosphorylation (Hu-Lowe et al., 2008) (Fig. S9C,D). Embryos were initially incubated with different concentrations (50 nM, 75 nM and 100 nM) of inhibitor to determine the optimal condition (75 nM) to induce reproducible phenotype without arresting development. Interestingly, although the expression patterns of VEGF ligands and receptors is strikingly similar to FGF components in embryos and regenerates in the brittle star (Fig. S9A,B), inhibition of the VEGF signalling pathway using Axitinib resulted in a much milder phenotype in respect to skeleton development in the embryos and regenerating explants of *A. filiformis* compared with the phenotype obtained with SU5402 treatment (compare Figs 2 and 3 with Fig. S9C,D). Axitinib-treated embryos usually formed one spicule during early development and this spicule elongated but failed to be patterned correctly (*n*=89/118) compared with normal skeletogenesis in FSW (*n*=102/119) and DMSO controls (*n*=101/123) (Fig. S9C). Only 36.6% of treated explants (*n*=41) showed reduced or absent spicules compared with 13.6% in DMSO controls (*n*=44) and 10% in FSW controls (*n*=40) (Fig. S9D). At the concentration used in this study, it is unlikely that SU5402 inhibition impinges significantly on the VEGF pathway, and specific inhibition of VEGF signalling shows that it is not strictly required for biomineralization to occur, but most likely for further patterning of the skeletal elements. We thus only focused on the molecular network affected by SU5402 treatment from this point on.

### Many genes downregulated by SU5402 are expressed specifically in skeleton-forming cells

**Fig. 5.**
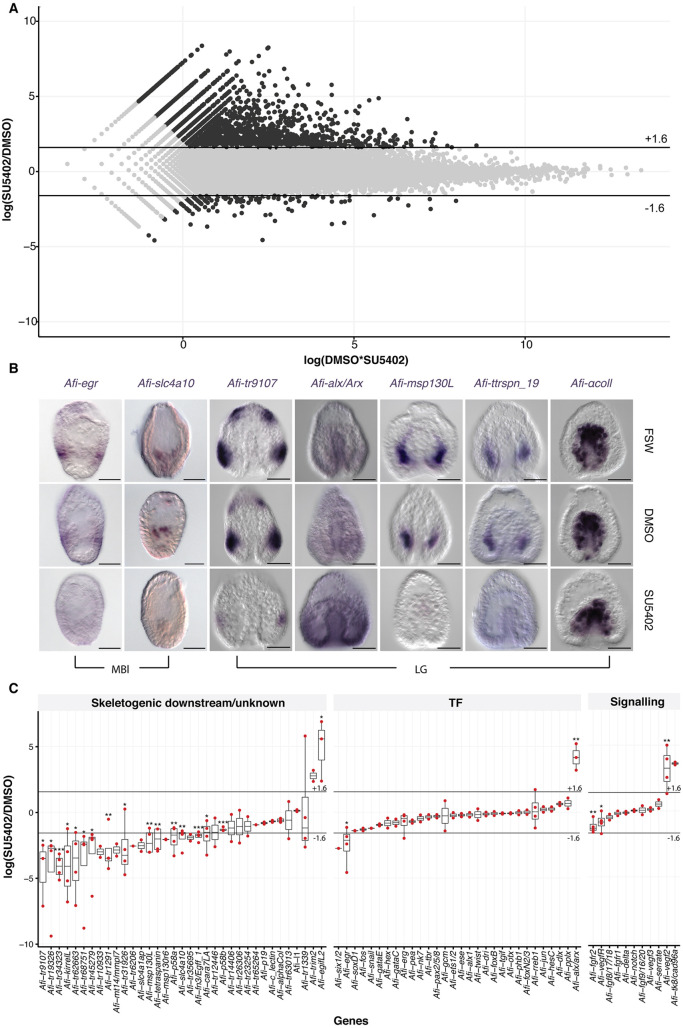
**Differential transcriptomic analysis and WMISH for SU5402-treated and control embryos.** (A) MA-plot showing upregulated (top) and downregulated (bottom) genes in response to SU5402 treatment. (B) WMISH on embryos treated with SU5402 that were fixed at gastrula stage. *Afi-αcoll* was used as negative control and no change in expression was observed. *Afi-egr*, *Afi-slc4a10*, *Afi-tetraspanin 19* (Afi-*ttrspn19*), *Afi-msp130L* and *Afi-tr9107* are downregulated and *Afi-alx/arx* is upregulated in SU5402-treated samples. Embryos are all oriented with apical pole at the top and vegetal pole at the bottom. (C) Box plot summarizing differential gene expression in SU5402-treated embryos relative to DMSO showing consistency between transcriptome, qPCR and NanoString quantification strategies represented as log2(SU5402/DMSO). Scale bars: 50 μm.

To identify putative skeletogenic genes and other unknown targets of the FGF signalling pathway, we conducted a transcriptome-wide analysis of SU5402-treated embryos relative to controls (Fig. 5). In this analysis we used a log fold change threshold of ±1.6 log2(SU5402/DMSO), as used for sea urchin (Wei et al., 2006; Weitzel, 2004), to select up- or downregulated candidates in the transcriptome dataset (Materna and Oliveri, 2008). With this threshold, we obtained 140 downregulated and 2366 upregulated transcripts (Fig. 5A). As SU5402 inhibited skeleton development (Fig. 2B), we focused our attention on the downregulated genes to pinpoint potential candidates that may be involved in skeleton formation. In the 140 downregulated transcripts, and using our transcriptome annotation (Dylus et al., 2018), we found 101 sea urchin homologs, of which only three were TFs [*Afi-six1/2* (*Afi-six1*), *Afi-egr* (*Afi-egr3*) and *Afi-soxD1* (*Afi-sox5*)] and 16 were known skeletogenic genes (Fig. 5C). To improve the power of our predictions and to validate the differential transcriptome analysis, we performed NanoString on 123 selected candidates (26/140 downregulated, 5/2366 upregulated and other genes potentially involved in regeneration and development of skeleton; Tables S4 and S5) on two biological replicates, and quantitative polymerase chain reaction (qPCR) on three biological replicates using a subset of these 123 candidates. In order to compare data across different technologies, quantitative data were collected on the same sample using all three technologies (RNA-seq, qPCR and NanoString) and used to identify conversion factors to bring all data from different biological replicates on a comparable quantitative scale (details in Materials and Methods and Fig. S10). With this approach we were also able to compute additional significance values: 24 genes showed significant differences (*P<0.05), of which 12 were below log2(fc) −1.6 and three were above +1.6, and the residual nine were close to ±1.6 (Fig. 5C). Interestingly, in the embryo only a few TFs were affected in the transcriptome-wide analysis and none of them are known TFs expressed in the SM. On the contrary, FGF and VEGF signalling components showed significant differential expression: both of the receptors specifically expressed in SM cells [*Afi-fgfr2* and *Afi-vegfR* (*Afi-flt1*)] are downregulated in SU5402-treated embryos, whereas the *Afi-vegf2* ligand is upregulated (although with its regular low expression this might be an artefact; Tables S2 and S3). To address the spatial expression of differentially expressed transcripts, we performed WMISH on selected genes from another biological replicate (Fig. 5B). WMISH on four downregulated transcripts specifically expressed in SM cells (*Afi-msp130L*, *Afi-tetraspanin19*, *Afi-tr9107*, *Afi-slc4a10*), and one ectodermally expressed gene *Afi-egr*, consistently showed loss of expression in SU5402-treated samples, whereas an increase in ubiquitous expression of *Afi-alx/arx* was detected, a gene identified as upregulated in all our quantitative expression assays. As a negative control the unaffected gene *Afi-αcoll* shows no change of expression (Fig. 5B). These data indicate that the combination of different technologies on different biological replicates resulted in a reliable list of candidate genes that were affected by SU5402.

### Molecular effects downstream of FGF signalling in embryogenesis and regeneration

To compare the genes transcriptionally regulated by FGF signalling between development and regeneration we performed a large-scale analysis of the effects of SU5402 perturbation in explants using NanoString. We used a code set of 123 genes and quantified three biological replicates of RNA extracted from 10 individual arm explants treated with SU5402 for 24 h (at stage 2) relative to controls. To detect differentially expressed candidates a log fold change of 1 was used as a threshold of significance, similar to previously published work (Cui et al., 2014). In this analysis we found 25 differentially expressed genes (10 upregulated and 15 downregulated). As many candidates were found to be close to a fold change of ±1, we additionally assessed statistical significance using the two-tailed unpaired Student's *t*-test. We found 23 differentially expressed genes, of which seven were shared between the threshold and *t*-test. Due to the small overlap, not even 50%, we compared the distributions of standard deviations between arms and embryos. We found a higher dispersion in the samples collected from arms than in the samples from embryos (Fig. S11). A possible explanation for such a high variance may be that arm samples are more heterogeneous and also contain only a small proportion of skeletogenic cells, thus increasing the noise-to-signal ratio and making it more difficult to find affected genes using standard quantitative approaches.

**Fig. 6.**
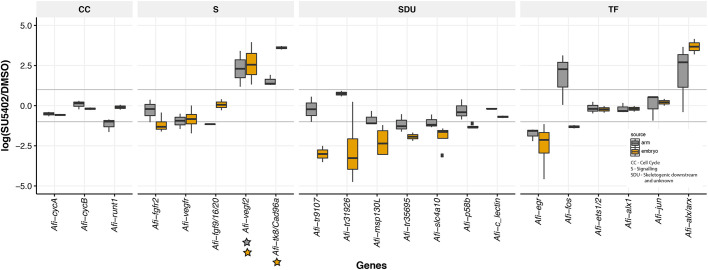
**Comparison of genes affected by FGF signalling perturbation in embryos and regenerating arms of *A. filiformis*.** Boxplot of selected genes showing the median and data distribution (box, interquartile range; whiskers, maximum and minimum expression value; horizontal line, median) of gene quantification obtained in SU5402-treated embryos (grey) and regenerates (yellow) relative to DMSO controls, from at least three biological replicas. The relative abundance is expressed in log2(SU5402/DMSO) and threshold is set at ±1 corresponding to 2-folds of difference (grey horizontal line). Genes have been divided in functional categories: CC, cell cycle; S, signalling; SDU, skeletogenic downstream and unknown; TF, transcription factors. Stars under a gene indicate very low level of expression in control embryos (yellow; see Table S2) or in regenerating arms (grey; see Table S3).

To address whether the molecular effects of FGF signalling on skeleton development are similar between embryonic development and arm regeneration, we quantitatively compared the expression of various genes in the two processes. Of the 123 genes quantified using NanoString technology, we found 24 in arm and 15 in embryo to be expressed below background (<20 counts comparable with internal negative control of the NanoString). Using the threshold of log2(SU5402/DMSO) ±1 for a better comparison, we found that overall 73 differentially expressed transcripts showed the same trend of expression between arms and embryos, with 59 downregulated (Fig. S12A) and 14 upregulated (Fig. S12B), with 22 genes showing a different trend of expression (Fig. S12C). We performed WMISH on at least three SU5402-treated explants and relative controls for each gene from a selected group of transcripts (Fig. S13) to validate our quantitative analysis (Fig. 6). Transcripts classified as downregulated, specifically *Afi-egr*, *Afi-msp130L* and *Afi-slc4a10*, showed loss of expression in their respective territories (Fig. S13). *Afi-p58b* consistently showed no change of expression quantitatively or qualitatively (Fig. S13). Interestingly, *Afi-msp130L* was not part of the overlapping genes in the quantitative dataset but clearly showed no expression in SU5402-treated arms nor embryos by WMISH (Fig. 5 and Fig. S13), suggesting that our approach may be too stringent to detect all downregulated genes, especially in the more heterogeneous context of arm regeneration. Notably, we did not observe any expression changes in cyclin genes [e.g. *cycA* (*ccna2*), *cycD*] in SU5402-treated regenerates, in agreement with the EdU analysis showing that cell proliferation was not affected (Fig. 4). In addition, three transcripts, homologs to the uncharacterised sea urchin tyrosine kinase *Afi-tk8/Cad96a*, *Afi-vegf2* and the homeodomain transcription factor *Afi-alx/arx*, were all upregulated in SU5402-treated embryos and adult regenerates (Fig. 6), although *Afi-vegf2* and *Afi-tk8/Cad96a* had very low, almost undetectable, expression levels in normal embryonic development (Table S2). Interestingly, a few genes differentially affected by SU5402 relative to controls in the adult regenerating arms, but not in the embryos (Fig. 6) were stem cell-related TFs such as *Afi*-*runt1* (*Afi-runx2*) and *Afi*-*fos* (*fosl*), and signalling genes belonging to other pathways (such as *Afi*-*serrate*). Importantly, upstream skeletogenic specification TFs [such as *Afi*-*alx1*, *Afi-ets1/2* (*Afi-ets1*) and *Afi-jun*], as well a few downstream skeletogenic genes (*Afi-p19*, *Afi*-*αcoll* and *Afi*-*c-lectin*) (Dylus et al., 2016, 2018) are unaffected by FGF signalling inhibition in both embryos and adults (Fig. 6 and Fig. S12). Notably, although the signalling genes *Afi-fgf9/16/20* and *Afi-fgfr2* are expressed in comparable cell types between regeneration and development (see Fig. 1), we found *Afi-fgfr2* to be downregulated only in the embryo and *Afi-fgf9/16/20* to be downregulated only in the arm, suggesting differences in regulatory processes activating the FGF signalling components during the two processes. Finally, in both embryos and regenerating arms, FGF signalling inhibition affected the expression of VEGF signalling genes: *Afi-vegf2* was upregulated, and *Afi-vegfr* was mildly downregulated. This suggests a potential mechanism of cross-talk between the two signalling pathways.

### A new set of SM-specific genes are found to be affected by FGF signalling inhibition

Impairing FGF signalling severely affects development and regeneration of the skeleton in *A. filiformis*. The data in the previous sections show that a large portion of known skeletogenic genes (such as *p58a*, *kirrelL*, *msp130L*) require FGF signalling to be expressed in SM cells, therefore the differential transcriptome analysis conducted on embryos treated with SU5402 can be used to identify novel downstream genes involved in the development of skeleton in *A. filiformis*. Indeed, among the 140 downregulated genes, many (27 genes) do not have a clear homolog in the sea urchin genome, used as reference for annotation (Table S6). BLAST analysis showed that a handful of these transcripts have similarities with hemichordates or cnidarian genes (Table S7), 11 of them were included in the NanoString codeset and analysed for their expression and response to FGF signalling inhibition in embryos and regenerating arms. Nine of these new *A. filiformis* genes showed a similar response to SU5402 exposure in both the embryo and regenerating arms (Fig. S12). Bioinformatic analysis on five of these novel genes revealed that *Afi-tr31926* and *Afi-tr35695* are unique to brittle stars (also found in *Ophiocoma wendtii*) with no similarity to other sequences within analysed echinoderms (BLAST using Echinobase/EchinoDB databases) or in other organisms (NCBI non-redundant database) (Table S7). Protein structure prediction using PredictProtein (Yachdav et al., 2014) and analysis of conserved domains using CDART and PFAM databases revealed that these genes are likely to be secreted (presence of a signal peptide) and one of those (*Afi-tr35695*) is predicted to have calcium ion binding activity, which would be consistent with its putative role in the formation of a calcium carbonate skeleton (Table S7). WMISH showed that *Afi-tr31926* and *Afi-tr35695* were indeed expressed in the skeletogenic mesoderm in both the embryo and in the regenerating arm, either in early stages, late stages or both (Fig. S14). *Afi-tr9107*, on the other hand*,* was expressed in the ectoderm in a pattern that was reminiscent of the expression of the signalling ligands *Afi-fgf9/16/20* and *Afi-vegf3* in the ectoderm of the embryo at the boundary with the endoderm (Fig. 5), adjacent to where the skeleton is deposited. During regeneration this transcript is expressed in vertebrae and spines of late regenerating adult arms (Fig. S14).

Interestingly, in our analysis we also found two new genes that have not been previously described to have expression in SM cells in sea urchin. One is the transcription factor *Afi-rreb1*, not consistently downregulated in different biological replicas, and the gene *Afi-cara7la* (also known as *Afi-cah2*; Fig. 5C) consistently downregulated in SU5402-treated embryos. Both are specifically expressed in the skeletogenic territory in both embryos and regenerating arms (Fig. S13) and constitute additional novel skeletogenic genes identified in this study.

Altogether, these data identify new genes downstream of FGF signalling and similarities in the molecular network driving skeletogenesis between embryonic development and adult arm regeneration, suggesting that they are functionally equivalent.

## DISCUSSION

### FGF signalling is required for skeleton formation in the brittle star and activates a cassette of biomineralization genes

In this work we show that both brittle star skeletal development and adult regeneration rely heavily on the presence of FGF signalling. The evidence for this is as follows: (1) the expression pattern of FGF and VEGF ligands and receptors during development and regeneration allows for the ectodermal-mesodermal tissue interaction, which has been shown to be crucial for skeletogenesis in sea urchin embryos (Röttinger et al., 2008; Adomako-Ankomah and Ettensohn, 2013; Duloquin et al., 2007; Erkenbrack and Petsios, 2017); (2) perturbation of this pathway using the universal pharmacological agent SU5402 resulted in complete inhibition of skeletal spicule formation in both adult arms and embryos; (3) FGF signalling inhibition specifically downregulated the expression of genes involved in biomineralization. Similar to what was suggested for sea urchins (Röttinger et al., 2008), we show that the role of FGF signalling during skeletogenesis in the brittle star appears to be confined to downstream differentiation of skeletogenic cells, as putative upstream TFs (e.g. *Afi-alx1*, *Afi-ets1/2*) are unaffected. The observed effect on skeletal downstream genes (e.g. *msp130*, *slc4a10*, *kirrelL*), rather than transcriptional regulators, suggests a role of FGF signalling primarily in the differentiation step of skeleton development rather than in specification.

Proteomic studies have revealed hundreds of proteins associated with both the sea urchin and brittle star skeletal matrices (Mann et al., 2010; Seaver and Livingston, 2015). Interestingly, FGF signalling perturbation downregulated only a subset of those skeletogenic differentiation genes, while having no effect on others (e.g. *Afi-p19*, *Afi-c-lectin*). Nevertheless, this subset of downregulated genes is essential for skeleton formation, as their collective downregulation results in the failure of the last checkpoint in the skeletogenesis network – deposition of the biomineralized skeleton by mesenchymal cells. Those include genes belonging to the carbonic anhydrase gene family (e.g. *cara7la*), implicated in calcium carbonate deposition in various organisms including sea urchins (Mann et al., 2008; Livingston et al., 2006) and molluscs (Mann et al., 2012), solute carrier proteins such as *slc4a10*, and mesenchymal surface glycoproteins like *msp130L* (Illies et al., 2002).

### Functional conservation of FGF signalling in embryonic and regenerative skeletogenesis

**Fig. 7.**
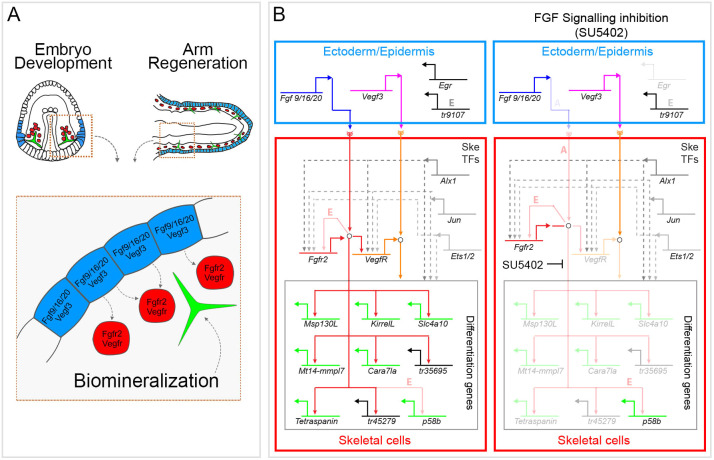
**Role of FGF signalling in skeletal cells in embryos and adult regenerating arms of *A. filiformis*.** (A) Top: skeletal cellular arrangement in a gastrula embryo and a stage 3/4 regenerating arm when biomineralized skeleton is deposited. In both cases mesenchymal cells (red) adjacent to ectodermal/epidermal cells (blue) secrete the biomineralized skeleton (green) in the extracellular space. Bottom: representation of the signalling occurring from ectodermal/epidermal to mesenchymal cells. (B) Left: hypothetical gene regulatory network for skeletal cells built with data coming from this work and previous publications (Dylus et al., 2016, 2018; Czarkwiani et al., 2013, 2016). Genes are colour-coded and are represented by their cis-regulatory control system: green are orthologues of genes known to be essential in the biomineralization process in sea urchin; genes of unknown function but known expression domain are in black. Genes are connected by functional linkages, which are either inferred (dashed lines) or confirmed (solid lines) in this study. Arrows indicate positive inputs (activation) and barred lines indicate negative inputs (repression). Open circles represent post-transcriptional/biochemical interactions occurring in the cytoplasm (phosphorylation of the FGF and VEGF receptors upon binding to the ligand and the complex intracellular cascade of signalling events). Right: representation of the same network in the presence of an FGF signalling inhibitor (SU5402). Downregulated genes are shown in shaded colours. E indicates linkages present only in developing embryos and A indicates linkages present only in regenerating arms.

A molecular conservation of genes expressed during embryonic development and regeneration has been previously shown in newts (Imokawa and Yoshizato, 1997) and chick embryos (Özpolat et al., 2012). However, these studies were limited to a comparison of only one or a few genes. Most recently, the transcriptomes of the embryo and regenerating stages of the sea anemone, *Nematostella vectensis*, have provided the first large-scale resource for comparing those two processes at a global level and also revealed important differences between them (Warner et al., 2018). It is thus of great interest to compare specific aspects of development between embryogenesis and regeneration, for example similar cell types or structures. Our previous work has already shown that the morphology and molecular signature of skeletogenic cells is highly similar between the embryo and regenerating adult arm of *A. filiformis* (Dylus et al., 2016; Czarkwiani et al., 2013, 2016). The importance of FGF signalling in skeleton development and regeneration in *A. filiformis* reveals additional functional similarities between skeletogenesis at both stages of the brittle star life cycle. Fig. 7 summarizes the underlying provisional molecular network downstream of FGF signalling in skeletal cells. It is highly conserved between regeneration and development, with several genes being specifically downregulated in both cases e.g. the biomineralization genes *Afi-kirrelL, Afi-msp130L* and *Afi-slc4a10*. A few key changes, however, are also revealed in our work: (1) differential response of FGF signalling components (*fgf19/16/*20 and *fgfr2*) to the SU5402 treatment in the two processes; (2) ectodermal expression of the gene *tr9107*; (3) the skeletal gene *p58*. Taken together, our data provide support for the hypothesis of regeneration re-capitulating development, at least at the level of cell differentiation, and provides a large-scale comparison of the molecular networks driving development and regeneration of the same cell type in the same species*.* It remains to be found whether the initiating molecular events upstream of this signalling pathway are also conserved or are significantly different, as suggested by other studies (Warner et al., 2019 preprint).

### Cross-talk between FGF and VEGF signalling regulatory networks

It has been previously suggested that the FGF and VEGF signalling pathways may function synergistically, whereby the downregulation of either of the ligands can affect the expression of the other pathway components (Adomako-Ankomah and Ettensohn, 2013; Tomanek et al., 2010). For example, specifically in sea urchins, downregulation of *fgf19/16/20* results in upregulation of *vegf3* expression, whereas downregulation of *vegf3* results in upregulation of *fgfr2* (Adomako-Ankomah and Ettensohn, 2013). Our analysis of downstream targets of the FGF pathway provides insights into the mechanisms of its transcriptional regulation in *A. filiformis*. Our results show that the inhibition of FGF signalling in skeletal cells impinges on the expression of VEGF pathway genes downregulating the receptor *Afi-vegfr*, expressed in skeletogenic cells. Moreover, SU5402 induces upregulation of the *Afi-vegf2* ligand gene that has very low expression in control embryos (Fig. S7). This is consistent with the very dramatic phenotype observed in the FGF inhibition experiments, which ultimately will also affect the VEGF signalling in these cells. This highlights how the two signalling pathways are interlinked not only in the sea urchin but also in the brittle star, albeit in a different manner. However, this presence of signalling cross talk underlines the difficulty with dissecting the roles of signalling pathways, which may be tightly linked to inter-regulatory and feedback loops, suggesting the presence of a signalling network in which ligands and receptors are under the control of other signalling pathways.

### Evolution of FGF signalling and skeleton formation in echinoderms

The evolution of the FGF gene family involved extensive gene duplication and gene loss, often lineage-specific (Oulion et al., 2012), resulting in complex and variable distribution of FGF genes among metazoans. In vertebrates, major duplications of the gene family occurred resulting in 19 FGFs in chicken and over 22 FGFs in mammals (Oulion et al., 2012; Ornitz and Itoh, 2015). There are far fewer receptors of the pathway, with only four functional FGFR genes in vertebrates (Itoh and Ornitz, 2004), two in sea urchins (Lapraz et al., 2006) and two in *Drosophila* (Itoh and Ornitz, 2004). Only one Fgf ligand has been described in sea urchins, whereas hemichordates have five ligands (Fan and Su, 2015), some of which result from specific duplications within the Ambulacraria. In *A. filiformis*, we identified two Fgf ligands and two Fgf receptors, suggesting that gene-independent duplication events from a common ancestral FGF ligand and receptor occurred in chordates as well as in echinoderms.

In sea urchin embryos, FGF signalling components are expressed in a complimentary pattern, whereby the *fgfr2* receptor is specifically expressed by the SM cells and the *fgf9/16/20* ligand is expressed in overlying ectoderm (Röttinger et al., 2008; Adomako-Ankomah and Ettensohn, 2013). Recent work has shown that this pattern of expression is also observed for the VEGF signalling genes in both sea urchin (Adomako-Ankomah and Ettensohn, 2013; Duloquin et al., 2007; Erkenbrack and Petsios, 2017) and brittle stars embryos, as well as in sea urchin and sea star juveniles (Morino et al., 2012; Gao et al., 2015). It has been suggested that the heterochronic activation of this pathway in sea urchin and brittle star embryos led to the co-option of the adult skeleton into the larva (Morino et al., 2012; Gao and Davidson, 2008), as sea star embryos do not have those genes expressed at the embryonic stage and have no larval skeleton (Morino et al., 2012). Our results show that both VEGF and FGF genes are expressed in a strikingly similar pattern in embryos and adult regenerating arms of *A. filiformis*, suggesting that the interaction of the skeletogenic cells with the ectoderm, mediated by those signalling pathways, may be a conserved feature for adult echinoderms, and has in fact been co-opted in the embryos of sea urchins and brittle stars to form a larval skeleton. Our data suggest that, in brittle stars, FGF signalling plays a more prominent role in skeletogenesis than VEGF signalling, which is the opposite case for sea urchins (Adomako-Ankomah and Ettensohn, 2013). Furthermore, the transcriptional regulation downstream of FGF signalling appears to be significantly different in brittle stars and sea urchins, namely: (1) ∼30% of genes identified in our differential screen did not have sea urchin homologs (e.g. *tr31926, tr35695*); (2) other genes with homologs are not specifically expressed in the skeletogenic lineage in the sea urchin (e.g. *Afi-rreb1*; Materna et al., 2006). Recent work has shown that, despite a striking similarity in the morphology and development of the larval skeleton in sea urchins and brittle stars, the dynamics of their regulatory states are very different, suggesting alternative re-wiring of the network in the two classes (Dylus et al., 2016). Together with our results showing the high degree of conservation of the brittle star embryonic and adult network downstream of FGF signalling, we can hypothesize that the embryonic program for skeletogenesis could have been independently co-opted in brittle stars and sea urchins. An alternative evolutionary scenario would imply a coordinated evolution of the skeletogenic program in larvae and adults. Elucidating the role of FGF signalling in adult skeletogenesis of the remaining four classes of extant echinoderms could help resolve this issue in the future.

### Evolutionary implications for skeletogenesis among deuterostomes

Skeletal regeneration is observed in other deuterostome groups: for example in cirri regeneration of amphioxus (Kaneto and Wada, 2011) and in appendage regeneration of different vertebrates (reviewed by Ferretti and Health, 2013). It has even been suggested that adult bone repair and regeneration may recapitulate embryonic bone development at a molecular level (Ferguson et al., 1999). Comparing the skeleton developmental program between embryogenesis and regeneration can be vital to understand the evolution of skeletogenesis in deuterostomes. Although the skeleton of echinoderms is composed of calcium carbonate, instead of calcium phosphate, similarities of its ontogeny can be observed when compared with vertebrates. For example, in both groups the trunk skeletal precursor cells are mesoderm-derived motile mesenchymal cells. Gene expression can also aid in understanding the extent of potential similarities. The key regulators of the sea urchin (and likely brittle star) skeletogenic GRN include TFs *alx1*, *ets1/2* and *erg* (Dylus et al., 2016; Czarkwiani et al., 2013; Ettensohn et al., 2003; Koga et al., 2010, 2016; Rizzo et al., 2006). Members of the Cart/Alx3/Alx4 group of TFs are also involved in skeletal development in vertebrates. They are expressed in embryonic lateral plate mesoderm, limb buds, cartilage and ectomesenchyme, and deletions of these genes result in cranial and vertebral malformations (Brouwer et al., 2003; ten Berge et al., 1998; Zhao et al., 1996). ETS family TFs (homologs of *ets1/2* and *erg*) have also been implicated in vertebrate skeletogenesis (Li et al., 2004; Raouf and Seth, 2000, 2002; Vlaeminck-Guillem et al., 2000; Kola et al., 1993; Iwamoto et al., 2007). FGF signalling has a highly conserved role in skeletogenesis in deuterostomes, as demonstrated in sea urchins (Röttinger et al., 2008), lampreys (Jandzik et al., 2014), chickens (Mina and Havens, 2007) and mice (Yu and Ornitz, 2008; Sarkar et al., 2001).

In terms of downstream biomineralization genes, the network has diverged significantly between echinoderms and vertebrates. Most of the biomineralization genes identified in sea urchins and brittle stars do not have apparent homologues in vertebrates or other invertebrate deuterostomes (Dylus et al., 2016; Seaver and Livingston, 2015; Livingston et al., 2006). Interestingly, the recent genome of the brachiopod *Lingula anatine*, which like distantly related vertebrates forms its shell using calcium phosphate, also reveals a unique expansion of a set of biomineralization genes (for example chitin synthases) different from duplication events which gave rise to bone formation genes in vertebrates (such as fibrillar collagens) (Luo et al., 2015). Those differences in the set of biomineralization genes used by brachiopods, echinoderms and vertebrates suggest that these animals independently evolved a core differentiation gene cassette via duplication events for building their calcium-based skeletons. Nevertheless, the initiation cascade, including the ancient signalling pathways (e.g. FGF, BMP) and TFs, appears to play a conserved role in these divergent animals (Livingston et al., 2006; Luo et al., 2015; Jackson et al., 2006, 2010; Murdock and Donoghue, 2011). Together with these studies, our work presents further evidence for an evolutionarily conserved regulatory apparatus driving the activation of biomineralization genes.

### Conclusions

In this study, we present a comparison of the role of FGF signalling in the embryonic development and adult regeneration of the skeleton in the brittle star *A. filiformis*. We characterized the expression of FGF and VEGF signalling pathway ligands and receptors during both embryonic development and adult arm regeneration. Using the inhibitor SU5402 we showed that perturbation of FGF signalling interferes with skeleton formation during both developmental processes. Our transcriptome-wide analysis of the effects of FGF signalling inhibition in brittle star embryos revealed a global view of the downstream targets of this pathway, including well-studied genes and novel brittle star skeletogenic genes. Finally, our comparative analysis of these FGF targets between embryos and adult regenerating arms strongly supports a high degree of conservation of the downstream molecular network underlying skeletogenesis. Although many processes are highly divergent between development and regeneration, such as wound healing and initial cellular organization, identification and comparison of the upstream signals activating the skeletogenic GRN in embryos and adults will elucidate whether regeneration truly re-capitulates development at the level of cell type specification and differentiation. This comparative work on skeletal development will also contribute to our understanding of the evolution of skeletogenesis within both echinoderms and deuterostomes more broadly.

## MATERIALS AND METHODS

### Adult animal maintenance and handling

Adult animals of *A. filiformis* were collected during their reproductive season (July-August) for embryo cultures and throughout the year for adult specimens in the Gullmarsfjord, Sweden in the proximity of the Sven Lovén Centre for Marine Sciences. Animals were maintained in the laboratory in London as described previously (Czarkwiani et al., 2013). Regenerating arm samples were obtained as described in Czarkwiani et al. (2016) and amputated arm explants were obtained as described in Burns et al., (2012). *A. filiformis* embryo culture was set up as previously described (Dupont et al., 2009). Treated and untreated embryos were collected at required stages for WMISH, RNA extraction and RNA-seq as previously described (Dylus et al., 2016, 2018). Arm regeneration experiments were conducted on animals of similar size, as an indication of similar age and with similar regeneration dynamics (Dupont and Thorndyke, 2006). Specifically, for non-regenerating arms one segment was cut from each arm. Similarly, for stage 1 arms at different time points (24 hpa, 48 hpa, 72 hpa) only the last segment before the amputated site was collected. For stages 3-5 only the regenerating bud was collected with no additional stump tissues. Finally, for the 50% DI stage arms, five segments from the proximal side of the regenerate closest to the stump and five segments from the distalmost side (excluding the distal cap) were collected corresponding to the most undifferentiated tissues (Fig. S6).

### Whole-mount *in situ* hybridization

The protocol for WMISH for embryos and adult regenerating arms of *A. filiformis* was identical except for the hybridization temperature as outlined below. The samples were first re-hydrated with graded ethanol washes (70%, 50% and 30%) and washed three times in 1× MA Buffer with Tween [MABT; 0.1 M maleic acid (pH 7.5), 0.15 M NaCl, 0.1% Tween-20] and pre-hybridized in hybridization buffer (HB) [50% deionized formamide, 10% polyethylene glycol, 0.05 M NaCl, 0.1% Tween-20, 0.005 M EDTA, 0.02 M Tris (pH 7.5), 0.1 mg/ml yeast tRNA, 1× Denhart's solution, DEPC-treated water] for 1 h at 50°C (regenerating arms) or 55°C (embryos). Next, the samples were put in HB containing 0.2 ng/µl antisense probe for 3-7 days at the same temperature. Following this period of time samples were post-hybridized in fresh HB without probe for 3 h, then washed once in MABT at the corresponding hybridization temperatures and once at room temperature (RT). The samples were then washed three times in 0.1× MABT, once more with 1× MABT before placing them in blocking solution (MABT, 0.5% goat serum) for 30 min. Samples were then incubated in 1:1000 anti-DIG AP (Roche, 11093274910) antibody solution overnight at 4°C. Next, the sample was washed five times in 1× MABT and twice in alkaline phosphatase (AP) buffer [Tris (pH 9.5), MgCl_2_, NaCl, Tween-20, levamisole, milliQ water] before adding the staining solution (AP buffer, 10% DMF, 2% NBT/BCIP) for the chromogenic detection. The staining was stopped with MABT washes.

### Inhibitor treatments and phenotypic analysis

SU5402 (Calbiochem) and Axitinib (Sigma-Aldrich) were dissolved in DMSO for a stock concentration of 10 mM and 5 mM, respectively. The drugs were added to embryo cultures at 18 hpf at a final concentration of 10µM (SU5402) and 75 nM (Axitinib), and the embryos were then allowed to develop until 27 hpf. At this time-point ∼500 treated and control (0.2% DMSO and FSW) embryos were collected for fixation for *in situ* hybridization and 500 embryos were collected for RNA extraction for qPCR and NanoString analysis. Remaining embryos were left to develop further for phenotypic assessment. Arm explants were used for testing the effects of inhibitors on regeneration and skeletogenesis. Adult *A. filiformis* arms were cut 1 cm from the disc and then left to regenerate until stage 2 (on average 5 dpa). Arms were then cut again 5 mm proximally to the initial amputation site to obtain explants, which were left for several hours to allow proximal wound healing. The explants were then incubated for 24 h in SU5402 at a final concentration of 10 µM or Axitinib at 200 nM. Samples reared in FSW and 0.1% DMSO were used as controls. The development of biomineralized skeletal primordia (or spicules) was monitored by incorporation of calcein (Sigma-Aldrich; 1:50 dilution of a 1.25 mg/ml stock solution), a green fluorescent dye that labels the newly deposited CaCO_3_ (Guss and Ettensohn, 1997). After the treatment, the arm explants were imaged for any morphological phenotype and fixed for WMISH or collected in RLT buffer (15 arms pooled together per condition) for NanoString analysis.

### Differential analysis of transcriptome data

Samples were quantified and normalized as previously described (Dylus et al., 2018). Differential analysis was conducted between SU5402- and DMSO-treated samples. As only one biological replicate was used for mRNA-seq, we used it to identify potentially differentially expressed candidates and validated those using other technologies. Candidates were selected based on two criteria: a user defined threshold of expression above 2 transcripts per million (tpm) (Nguyen et al., 2015) and a fold-change threshold of ±1.6 (Altenhoff et al., 2019).

As all three methods (transcriptome quantification, NanoString and qPCR) employ technologically different quantification strategies, we assessed their technical similarity by comparing fold change values of the different methods on the same biological replicate. Consistently, all three technologies showed a similar trend in fold change (88.1%). Transcriptome and NanoString fold change values for 114 genes showed high positive correlation (∼0.85) and linear regression analysis resulted in a significant positive association between the two techniques [β=1.12, 95% CI (0.97, 1.24), ***P<0.001, adjusted R^2^=0.7223; Fig. S6]. Interestingly, fold change values appeared to be generally slightly inflated in the transcriptome dataset (slope>1). When comparing fold change values of 31 genes between qPCR and transcriptome quantification we found a positive correlation (∼0.854) and that both techniques are positively associated [β=1.4340, 95% CI (1.10, 1.77), ***P<0.001, adjusted R^2^=0.7203; Fig. S6]. This is consistent with our observation comparing correlations of time-course datasets quantified using transcriptomics, NanoString and qPCR (Dylus et al., 2018). Importantly, as every technology encompasses differences in their technical error, we used the β and *y*-intersect values of the linear regression analysis to compare biological replicates across technologies.

### Inference of phylogenetic gene trees

For phylogenetic gene trees, sequences were collected from local assemblies and publicly available datasets (41 species). To fish out genes that contained the FGF, FGFR, VEGF and VEGFR domains, we obtained HMM profiles from the PFAM database. The sequences of the 41 species were scanned against these domains and were used to generate input data for OMA (v2.2.0) (Altenhoff et al., 2018). Hierarchical orthologous groups that contained our candidates were merged with groups that showed close blast similarity and were selected for further analysis. The merging step was necessary due to the independent divergence between chordates and echinoderms of more than 500 million years ago and still too low taxonomic sampling. Mafft (v7) (Katoh et al., 2017) was used for multiple sequence alignment, followed by several manual rounds of sequence trimming using maxAlign (v1.1) (Gouveia-Oliveira et al., 2007) or independent criteria such as retention of close sequence length to given candidate. For tree inference we used Iqtree (v.1.5.5) with LG model and 1000 fast bootstraps (Nguyen et al., 2015).

### Validation of differentially expressed candidates using qPCR and NanoString

To validate candidates obtained from the transcriptome analysis we performed a linear regression analysis between transcriptome versus qPCR and transcriptome versus NanoString using R. Coefficients obtained for slope and *y*-intercept were used to scale qPCR and NanoString samples in relation to the transcriptome. In this way, we accommodated differences in intrinsic technical errors of the various technologies.

### qPCR and NanoString nCounter analysis

qPCR analysis was performed as described previously for adult regenerating arms (Czarkwiani et al., 2013) and embryonic samples (Dylus et al., 2016). In addition, differential expression of genes was measured using the NanoString nCounter analysis system (NanoString Technologies) (Geiss et al., 2008). A 123-probe code set was designed based on *A. filiformis* sequences, including six different internal standard genes and a GFP probe for detecting spike-in GFP RNA (Table S4). For each experimental sample 100 ng of total RNA was used, extracted from 300 embryos or 10 regenerating arms using the RNeasy Micro Kit (Qiagen). Detected counts/100 ng of total RNA were normalized first using the positive control lane normalization provided in the NanoString nCounter cartridge and then again using selected six internal standard genes (normalization factor obtained using geometric mean for each lane). For quantifying differential gene expression in perturbed samples, a log2 fold change between controls and treated samples was calculated. The log2(SU5402/DMSO) of ±1 (reflecting a 2-fold difference in change of level of expression) was determined to be biologically significant in correspondence with previously published work (Cui et al., 2014).

### Cell proliferation assay

Regenerates treated with the SU5402 inhibitor were tested for changes in cell proliferation. The cell proliferation assay was carried out using the Click-iT^®^ EdU HCS Assay (Invitrogen) as described previously (Czarkwiani et al., 2016) and then imaged using confocal microscopy. For each regenerate between ∼100±10 slices were taken per *z*-stack (1 μm thickness). DAPI-labelled nuclei and EdU-labelled nuclei per stack were counted automatically using the Fiji plugin TrackMate (Tinevez et al., 2016). The number of EdU-labelled nuclei per total number of nuclei ranged from 672/3375 to 1385/5205. The proportion of nuclei labelled with EdU compared with all nuclei labelled with DAPI was calculated as a percentage. Two-tailed unpaired Student's *t*-test was used and showed no significant difference between control (DMSO) and SU5402-treated samples (*t*-value=0.261; *P*>0.25).

## Supplementary Material

Supplementary information
