# Supplementary material for: FGF signalling plays similar roles in development and regeneration of the skeleton in the brittle star Amphiura filiformis
Source: Development. 2021 May 27;148(10):dev180760. doi: 10.1242/dev.180760 (PMC8180256; doi:10.1242/dev.180760)
Supplement: Supplementary information [file develop-148-180760-s1.pdf]

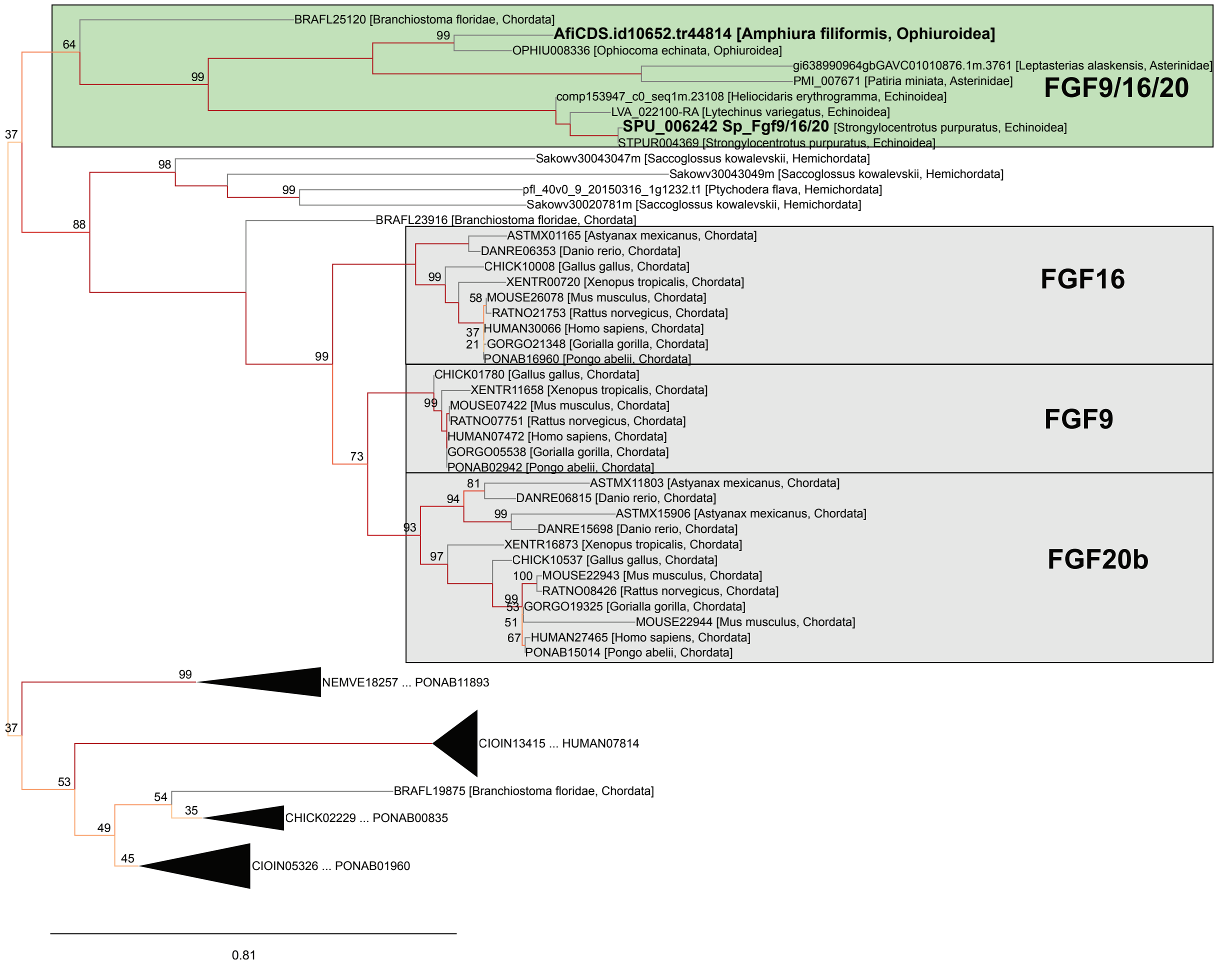

Figure S1: Tree for FGF9/16/20 genes. Maximum likelihood (ML) tree inferred using Iqtree (v.1.5.5) with LG model and 1000 fast bootstraps (1) with sequences derived from OMA group of FGF9/16/20 compute on 41 species (2,3). *Afi-fgf9/16/20,* along with other echinoderm FGF sequences, forms a well-supported group with the sea urchin *Sp-fgf9/16/20* gene (bootstrap 99), therefore considered an orthologous. The echinoderm *fgf9/16/20* genes form group related to vertebrate *FGF9*, *FGF16* and *FGF20* genes. On nodes are fast bootstrap values (1).

Development • Supplementary information

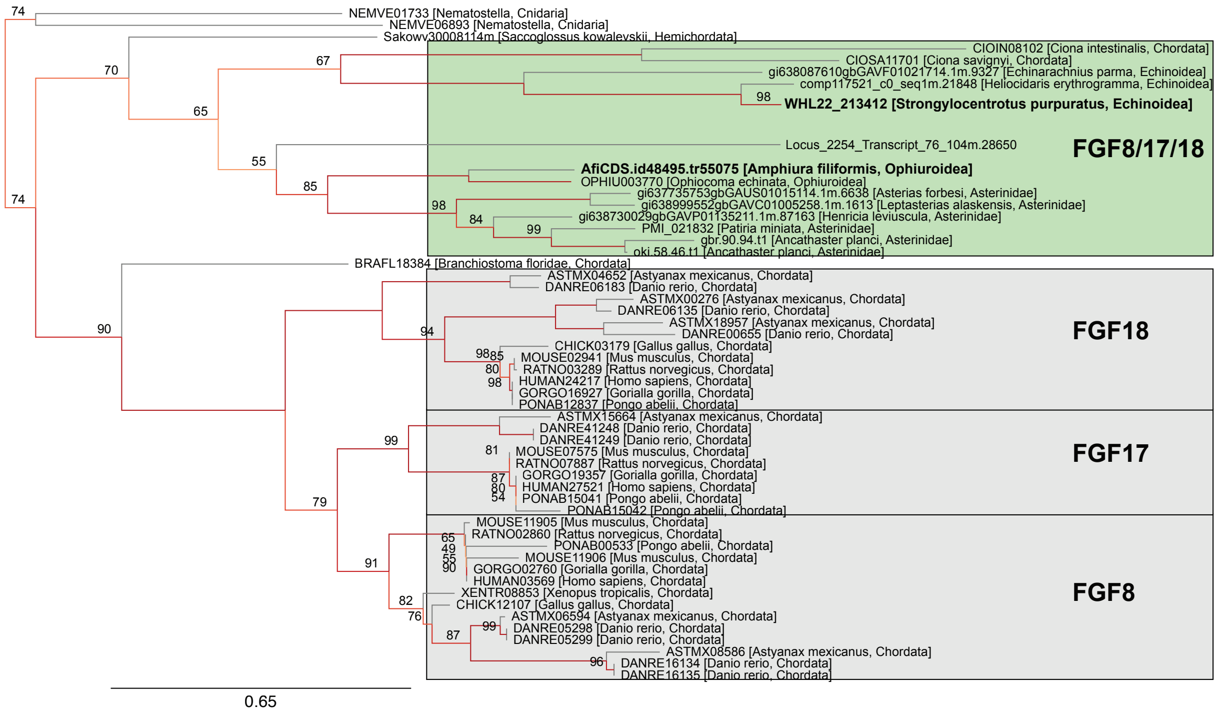

Figure S2: Gene tree for FGF8/17/18 ligands. ML Tree inferred with sequences derived from OMA group of FGF8/17/18 compute on 41 species (1, 2, 3). *Afi-fgf8/17/18* forms a group with a sea urchin sequence identified in the *S. purpuratus* transcriptome and the *S. kowalevskii fgf8/17/18* gene (bootstrap 70;(3)). This group has a clear relation to the group of chordate *FGF8*, *FGF17* and *FGF18* genes. On nodes are fast bootstrap values (1).

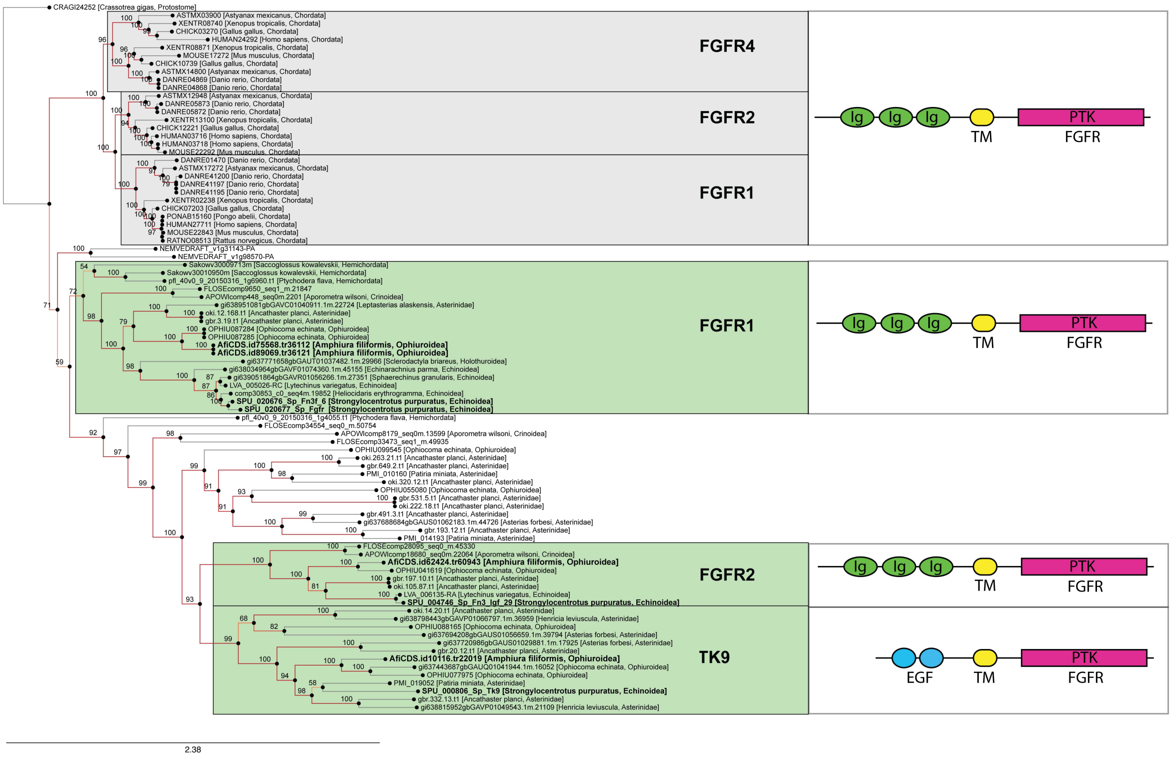

Figure S3: Gene tree for FGF receptors. ML tree inferred with sequences derived from OMA group of FGF receptors compute on 41 species (1, 2, 3). Three protein tyrosine kinase (PTK) sequences of the FGF receptor type have been identified in the *A. filiformis* transcriptome and here analysed along sea urchin, echinoderms and vertebrates sequences. *Afi-fgfr1*, *Afi-fgfr2* and *Afi-tk9* form well-supported groups with the *S. purpuratus* respective sequences (bootstrap of 99 and 100), therefore are considered orthologs to the sea urchin genes. All the echinoderms PTK in the tree are weakly related to the group of vertebrates FGFR 2, FGFR4 and FGFR1. The tree topology suggests independent duplication in echinoderms and in vertebrates of *fgfr* genes from a common gene in metazoans. On the right are schematically reported the protein conserved domain analyses that show the presence of PTK and trans membrane domains (TM) in each of the *A. filiformis* sequence, however only *Afi-fgfr1*, *Afi-fgfr2* encode for the three immunoglobulin extracellular domains (Ig) necessary to bind FGF ligands. On the contrary, the *Afi-tk9* sequence encodes for two extracellular epidermal growth factor (EGF) domains typical of other classes of PTK receptors, therefore we consider the *Afi-tk9* not a FGF receptor. On nodes are fast bootstrap values (1).

Development • Supplementary information

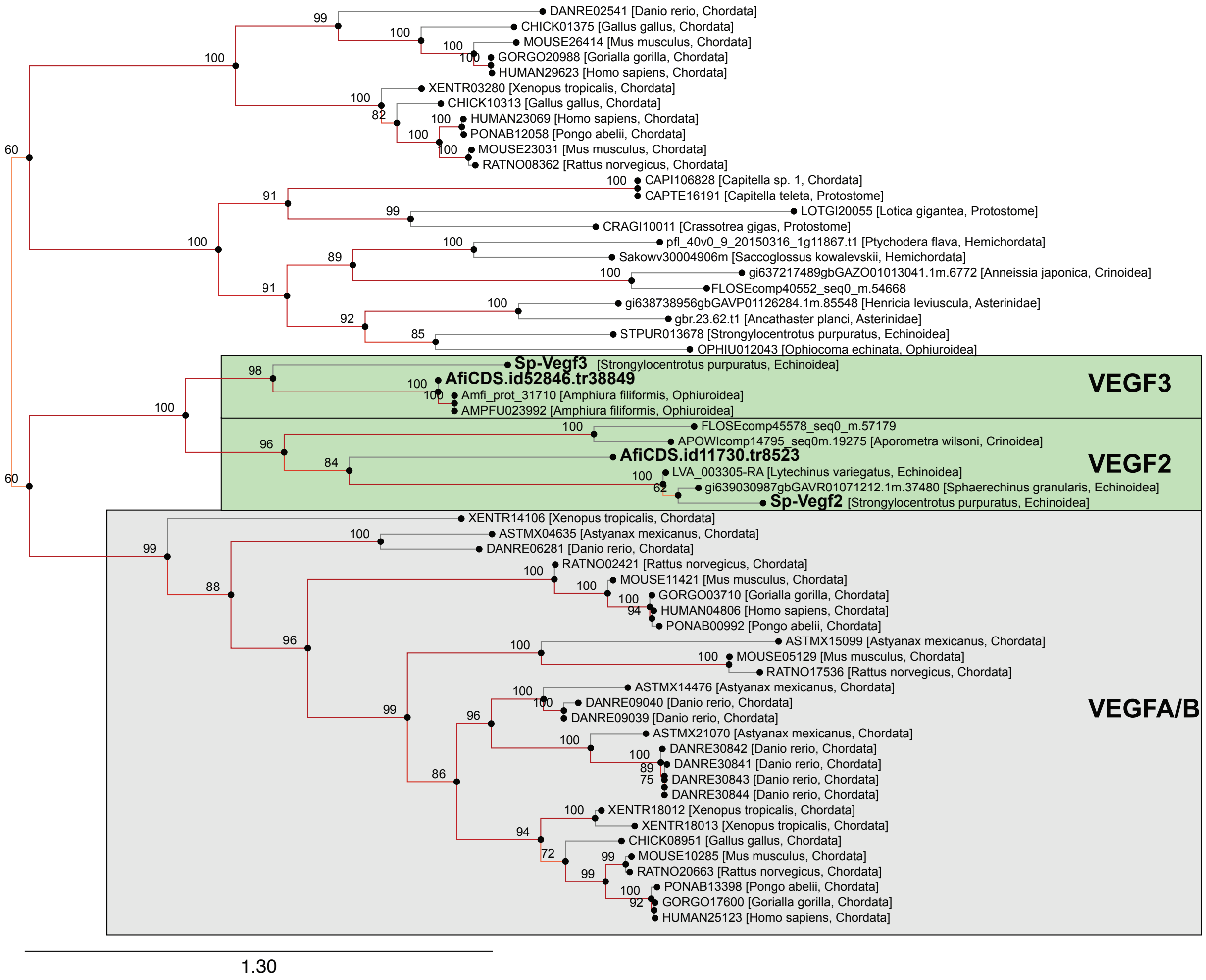

Figure S4: Gene tree for VEGF ligands. ML Tree inferred with sequences derived from OMA group of VEGF ligands compute on 41 species (1, 2, 3). *Afi-vegf2* and *Afi-vegf3* form well-supported groups with the sea urchin and other echinoderms *vegf2* and *vegf3* (bootstrap 96 and 98 respectively) genes, we therefore consider them orthologs to their sea urchin counterparts. The tree suggests that both ligand genes are descendants from an ancestral *vegf* gene that got independently duplicated in chordates. On nodes are fast bootstrap values (1).

Development • Supplementary information

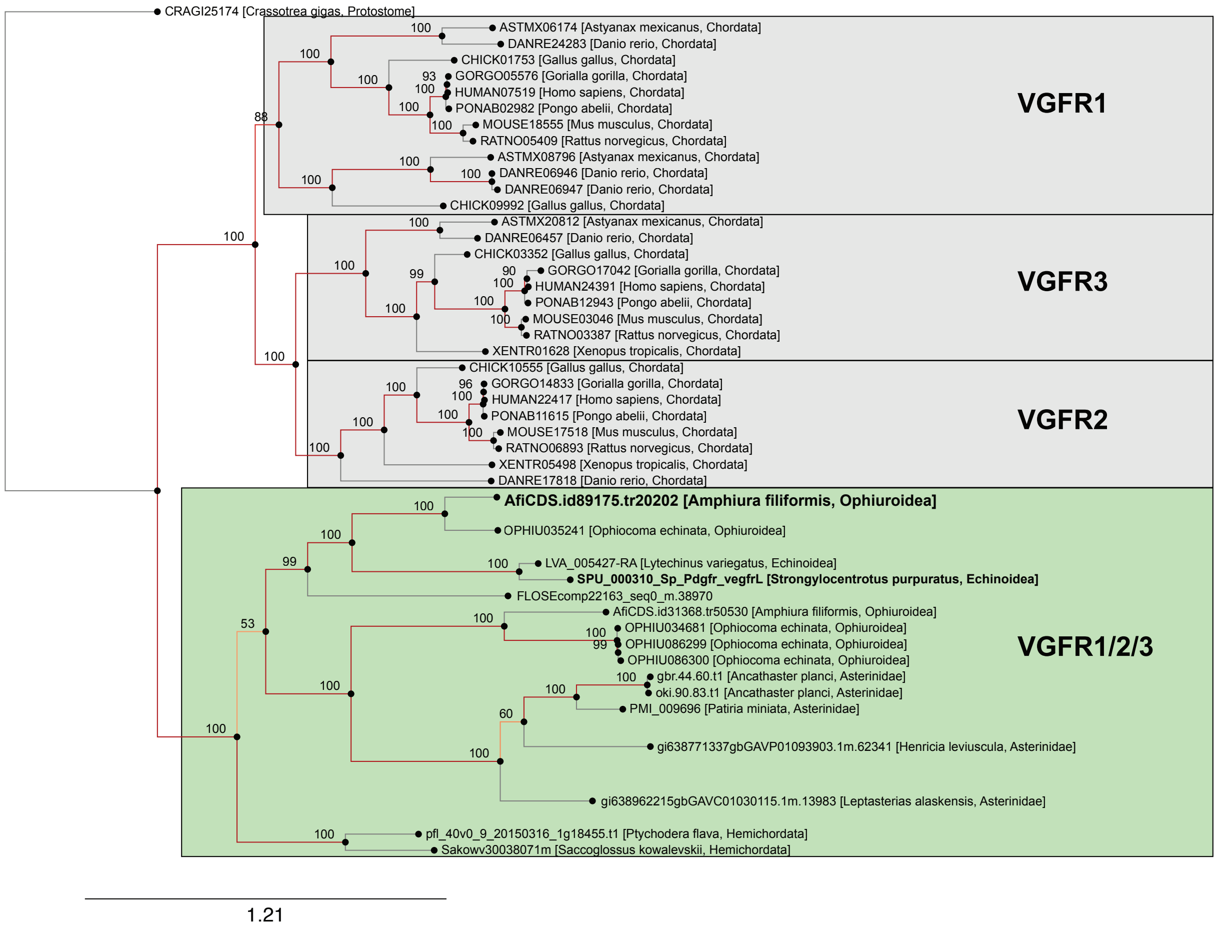

Figure S5: Gene tree for VEGF receptor. ML Tree inferred with sequences derived from OMA group of VEGF receptors compute on 41 species (1, 2, 3). A single Vegf receptor sequence has been identified in the *A. filiformis* transcriptome. *Afi-vegfr* groups to *S. purpuratus vegfr* gene (bootstrap 99) and is related to the chordate group of *Vegfr* genes (VEGFR1, VEGFR2 and VEGFR3). On nodes are fast bootstrap values (1).

Development • Supplementary information

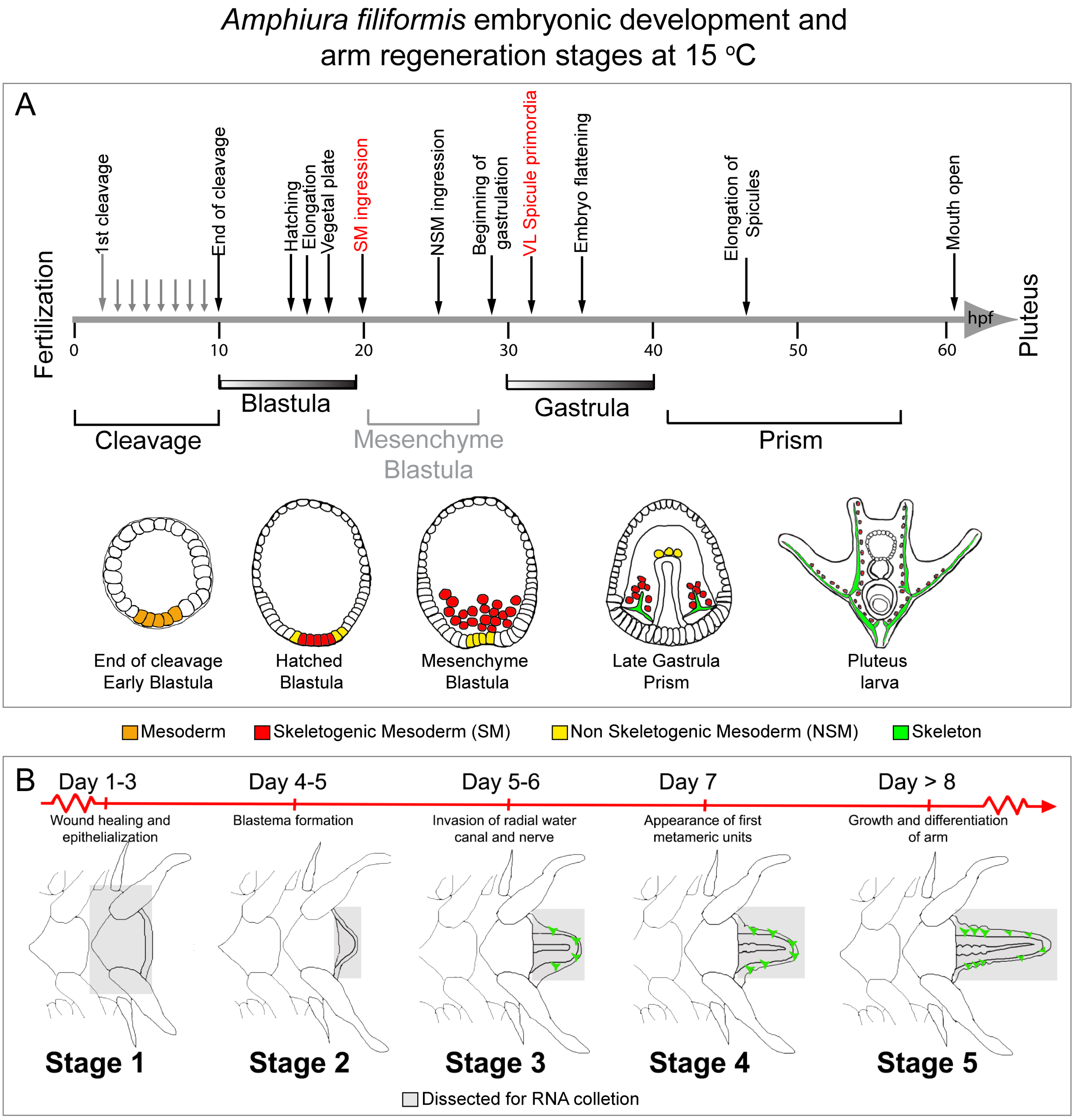

Figure S6: *A. filiformis* developmental and regeneration timeline. A) Embryonic timeline in hours post fertilization (hpf) with highlighted major event occurring, the specification of the skeletogenic mesodermal (SM) lineage and the formation of biomineralized skeleton(4). B) Timeline of major regenerative events and staging system expressed in days post amputation (dpa)(5). The wound healing, epithelialization and the absence of active cell proliferation characterize stage 1. At Stage 2 cell proliferation became very prominent and a small regenerative bud (or blastema) is visible. Stage 3 is characterized by a well-structured regenerative bud with clear regenerating radial water canal (RWC), radial nerve chord (RNC) and the appearance of skeleton primordia. Stage 4 shows the formation of the first metameric unit in most proximal position, while Stage 5 is considered when several metameric units are visible.

Development • Supplementary information

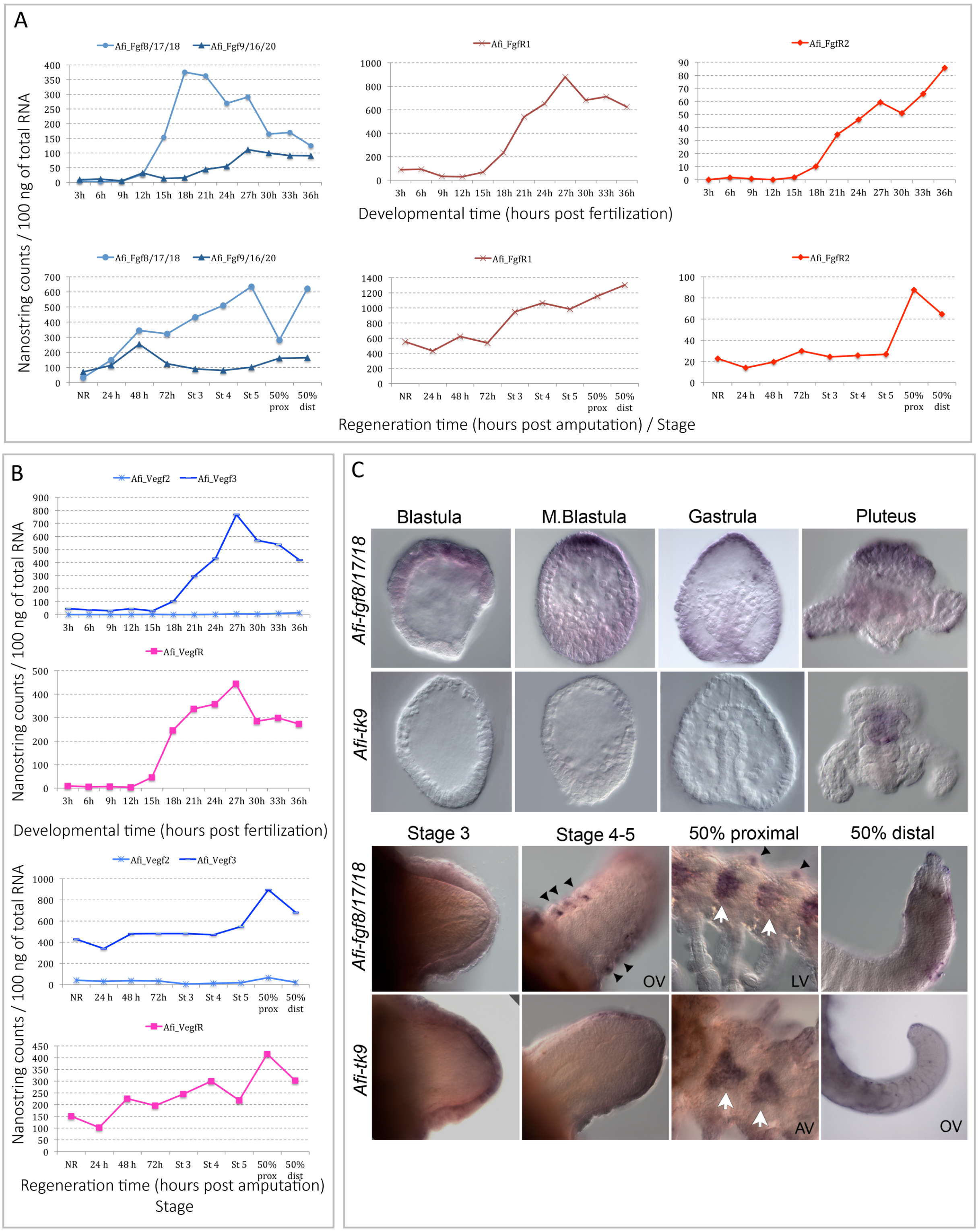

Figure S7: Expression profiles of FGF and VEGF signalling component genes during embryonic development and arm regeneration in *A. filiformis*. A) Levels of expression of *Afi-fgf9/16/20*, *Afi-fgf8/17/18*, *Afi-fgfr1* and *Afi-fgfr2* in embryos and adult non-regenerating and regenerating arms at different stages. B) Expression profiles of *Afi-vegf2*, *Afi-vegf3*, and *Afi-vegfr* in embryos and adult non-regenerating and regenerating arms at different stages. Transcript abundance is represented as

Development • Supplementary information

NanoString counts per 100ng of Total RNA. Hpf – hours post fertilization, hpa – hours post amputation, prox – proximal, dist – distal, NR – non-regenerating. C) WMISH on *Afi-fgf8/17/18* and *Afi-tk9* at different embryonic developmental stages and in early and late stages of adult arm regeneration in *A. filiformis*. *Afi-fgf8/17/18* shows expression in the apical region of the ectoderm throughout all the embryonic stages analysed and in the apical organ of the pluteus larva. In the regenerates WMISH identifies specific expression in spines at stage 4/5 and in late regeneration also in the vertebras. *Afi-tk9* shows no expression during embryonic development. Specific staining is identified in the foregut of the larva. In the early regenerating stages and the distal tip of the late regenerates the probe for *Afi-tk9* stains specifically the epidermis, while in proximal elements the vertebras are also showing expression. White arrows – expression in vertebrae, Black arrowheads – expression in spines. LV – lateral view, AV – aboral view, OV – oral view. Embryos are all oriented with apical pole at the top and vegetal pole at the bottom; regenerating arms are oriented with proximal on the left and distal on the right.

Development • Supplementary information

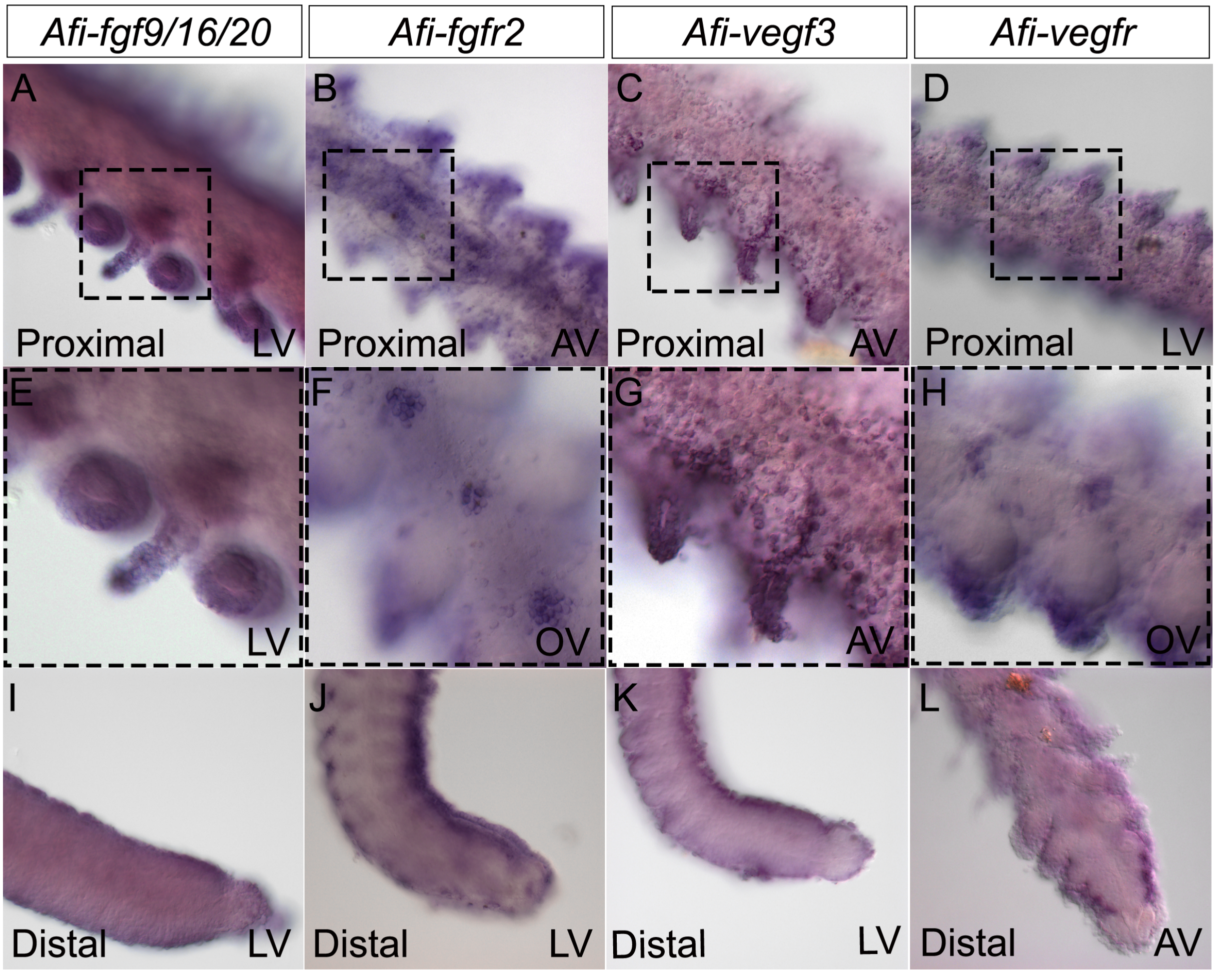

Figure S8: Expression of FGF and VEGF genes at late stages of arm regeneration in *A. filiformis*. WMISH conducted in 50% regenerative arms reveal: *Afi-fgf9/16/20* (A, E and I) still expressed in the epidermis both of proximal and distal structures. *Afi-fgfr2* (B,F and J) probe detects the developing skeletal elements such as vertebras and lateral shields in the proximal, most developed, part of the regenerates, while in the distal tip the staining is confined to the dermal cells. Similarly, the *Afi-vegf3* (C, G and K) is detected epidermal structured throughout the late regenerate, while the *Afi-vegfr* is revealed in developing skeletal elements and dermal cells. Av – aboral view, OV – oral view, LV – lateral view. Regenerating arms are oriented with proximal on the left and distal on the right.

Development • Supplementary information

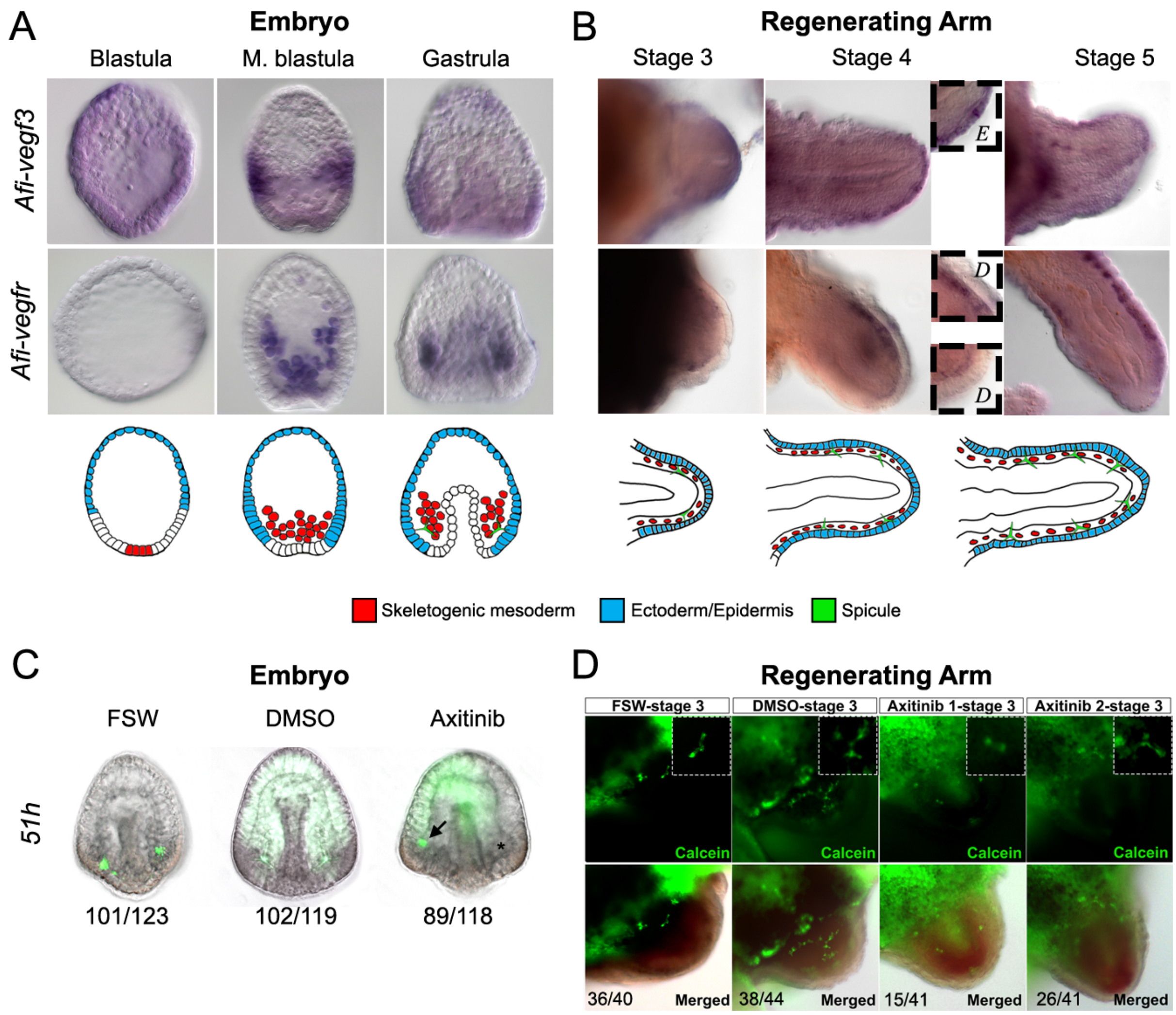

Figure S9: Expression of VEGF signalling components and axitinib treatment in embryos and early regenerating arm stages of *A. filiformis*. A) Top: WMISH on embryos at blastula, mesenchyme blastula and gastrula stages of development showing the expression of *Afi-vegf3* and *Afi-vegfr*. Bottom: schematic diagram of major relevant cellular domains. B) Top: WMISH on regenerates at stages 3, 4 and 5 showing the expression of *Afi-vegf3* and *Afi-vegfr*. Insets show detail of expression patterns. Bottom: Schematic diagram of major relevant cellular domains in regenerates. C) Phenotypic analysis of axitinib-treated embryos (75 nM) and controls at 51 hpf shows that perturbation of VEGF signalling results in embryos with one skeletal spicule forming. Numbers at the bottom show counts for embryos observed with the represented phenotype/total embryos counted. D) Phenotypic analysis of axitinib-treated regenerates and controls at 24 hours post treatment (stage 3) show that perturbation of VEGF signalling either results in normal or slightly reduced skeletal spicules. Numbers at the bottom show counts for explants observed with the represented phenotype/total explants counted. Insets show magnification of skeletal phenotypes. Embryos are all oriented with apical pole at the top and vegetal pole at the bottom; regenerating arms are oriented with proximal on the left and distal on the right.

Development • Supplementary information

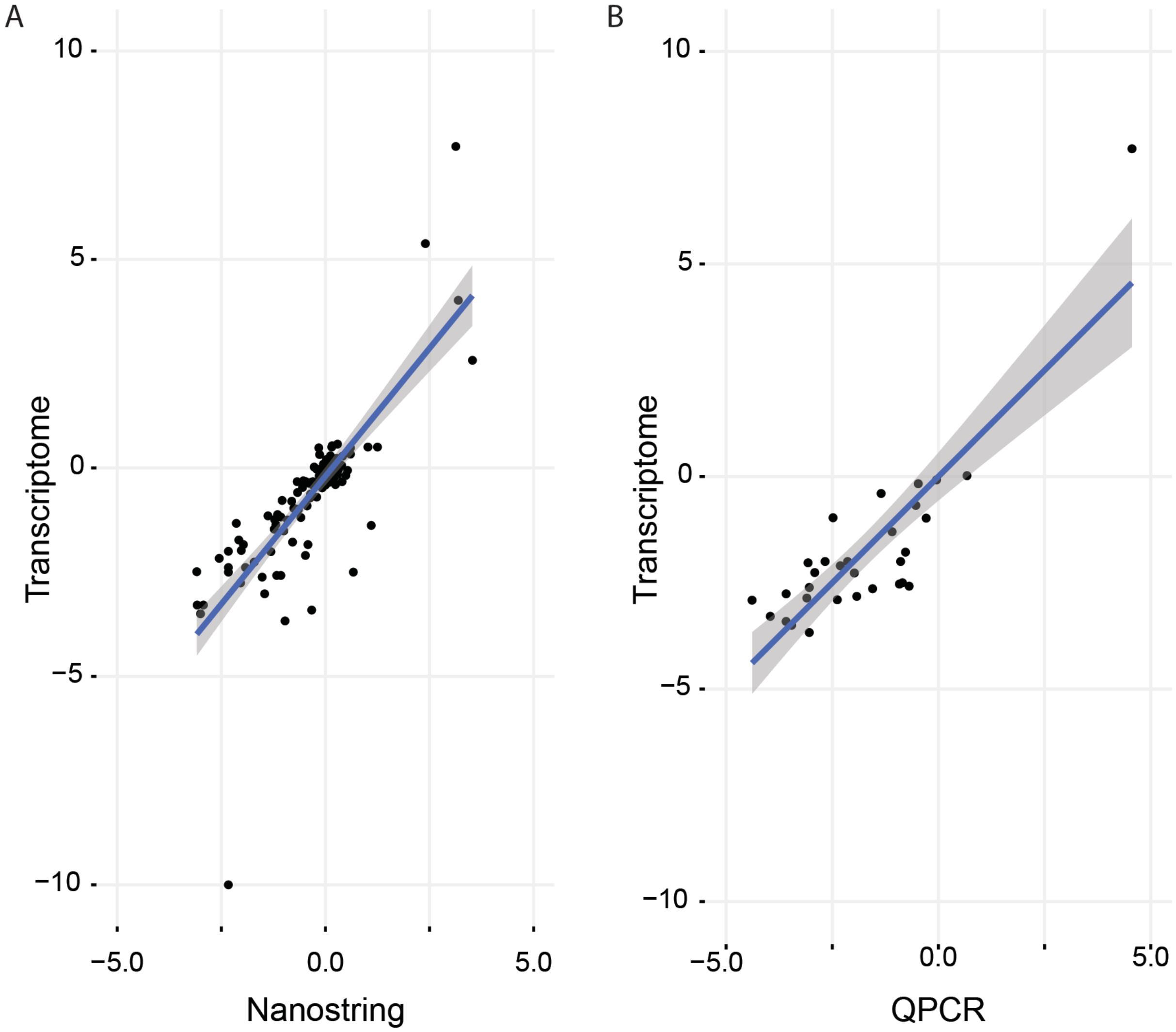

Fig S10: Comparison of differential expression values obtained from quantitative data collected on the same sample using all three technologies (RNA-seq, QPCR and NanoString). Linear regression was used to identify conversion factors as means to bring all data from different biological replicates on a comparable quantitative scale (Transcriptome). A) Comparison between Transcriptome and NanoString quantification strategies in embryos of *A. filiformis*. A significant linear regression was found ($F(1,111)= 292.3$, p-value: $< 2.2e-16$), with an R2 of 0.7247. B) Comparison of Transcriptome and QPCR quantification strategies in same embryo RNA samples of *A. filiformis*. A significant linear regression was found ($F(1,29)= 78.25$, p-value: $<9.877e-10$), with an R2 of 0.7203. Details are available in Methods.

Development • Supplementary information

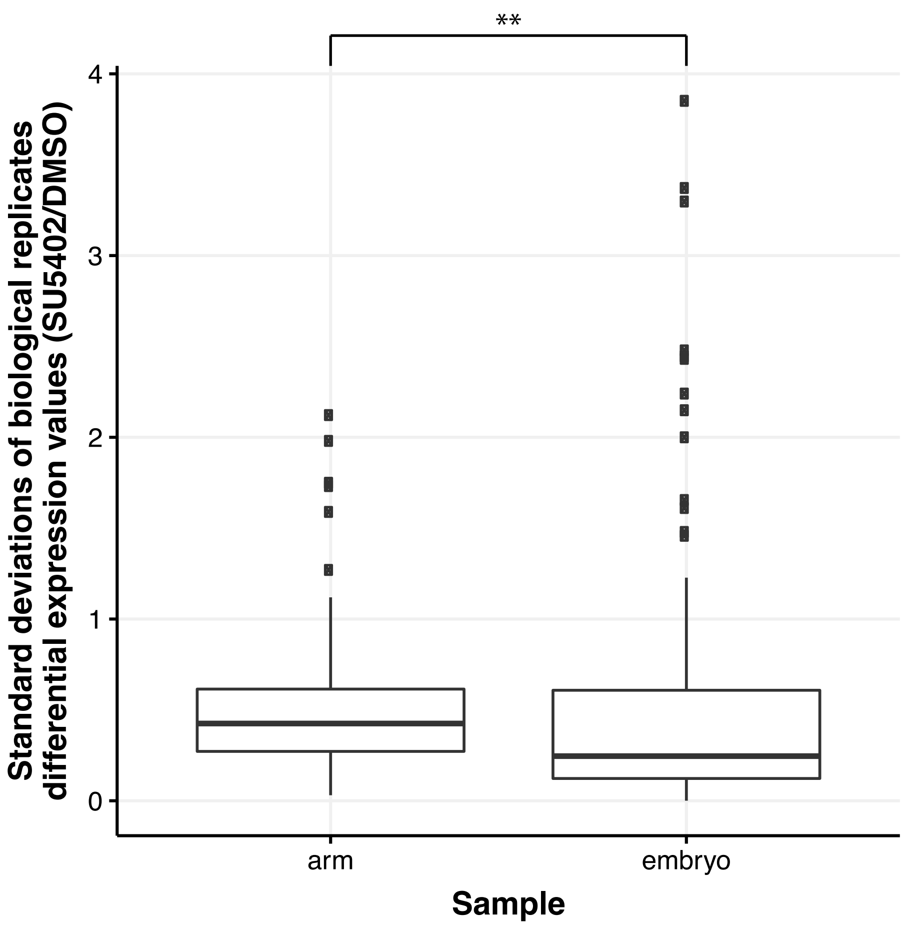

Figure S11: Standard deviations of differential expression are significantly lower in embryos than in arms. Boxplot was obtained using standard deviation values taken from at least 3 biological replicates for arm and embryo respectively as presented in Fig S12. A higher median in arm indicates that individual replicates in arms are displaying a higher dispersion than in embryos. This difference is significant (Wilcox-rank test p-value=0.002329).

Development • Supplementary information

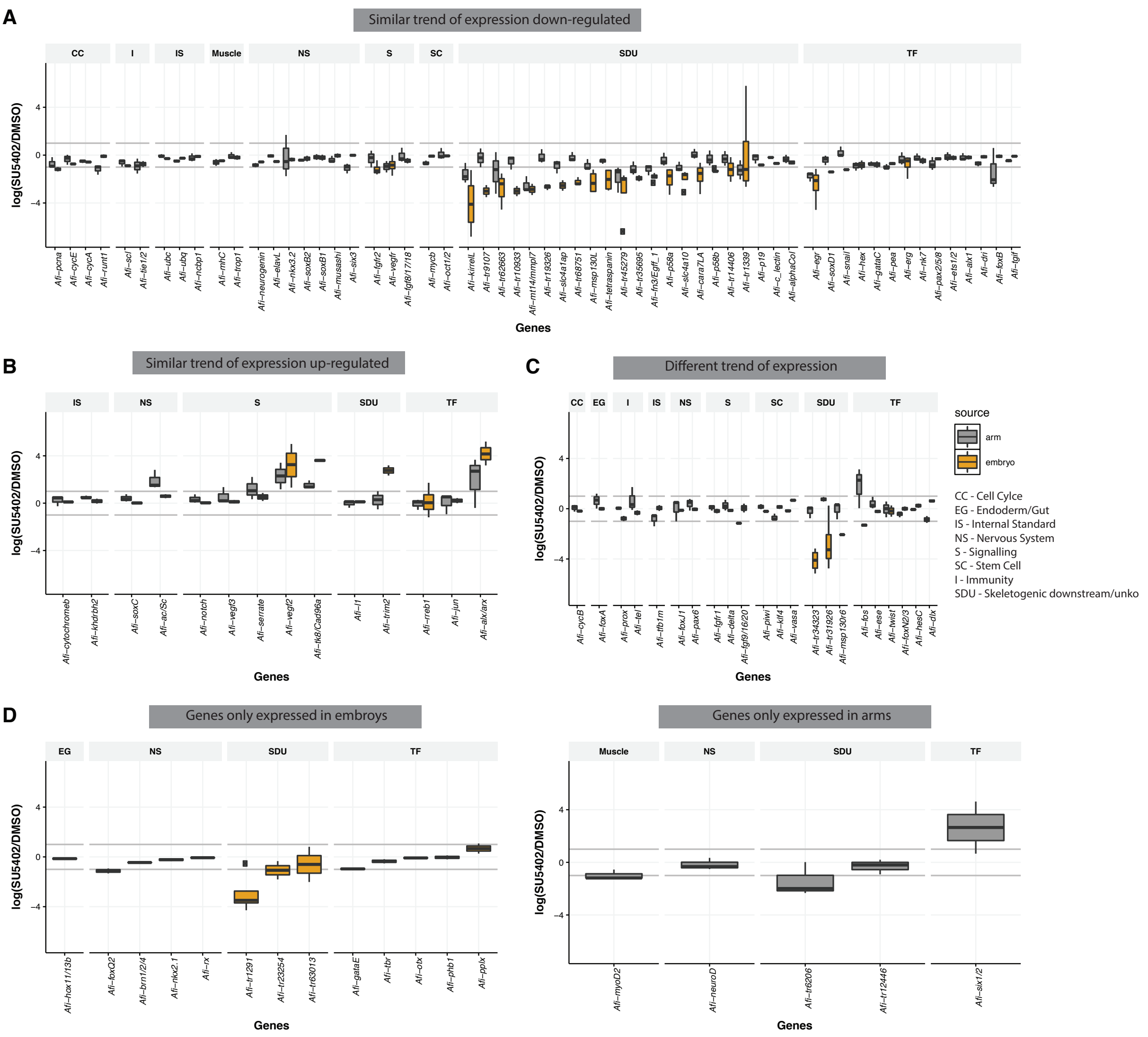

Figure S12: Comparison of genes affected by SU5402 treatment in embryos and regenerating arms of the brittle star. Boxplot showing the median and data distribution of at least 3 biological replicates across different technologies (analysed as described in methods) of gene quantification in SU5402 treated embryos (grey) and regenerates (yellow) relative to DMSO controls. A) Downregulated genes that show similar trends between embryos and regenerates; B) similar trends in upregulated genes. C) Genes that have opposite trend in expression between embryos and regenerates. In D) are genes expressed above background levels exclusively during embryonic development; and E) only during regeneration. The relative abundance is expressed in log2(SU5402/DMSO) and threshold is set at ±1 log2(SU5402/DMSO) corresponding to 2-folds of difference (grey horizontal line). Genes downregulated in the SU5402 treatment have negative values and genes upregulated positive. Genes have been divided in functional categories: CC – cell cycle; EG – endoderm/gut; IS – internal standard; NS – nervous system; S – signalling; SC – stem cells; I – immunity; SDU – skeletogenic downstream/unknown; TF – transcription factors.

Development • Supplementary information

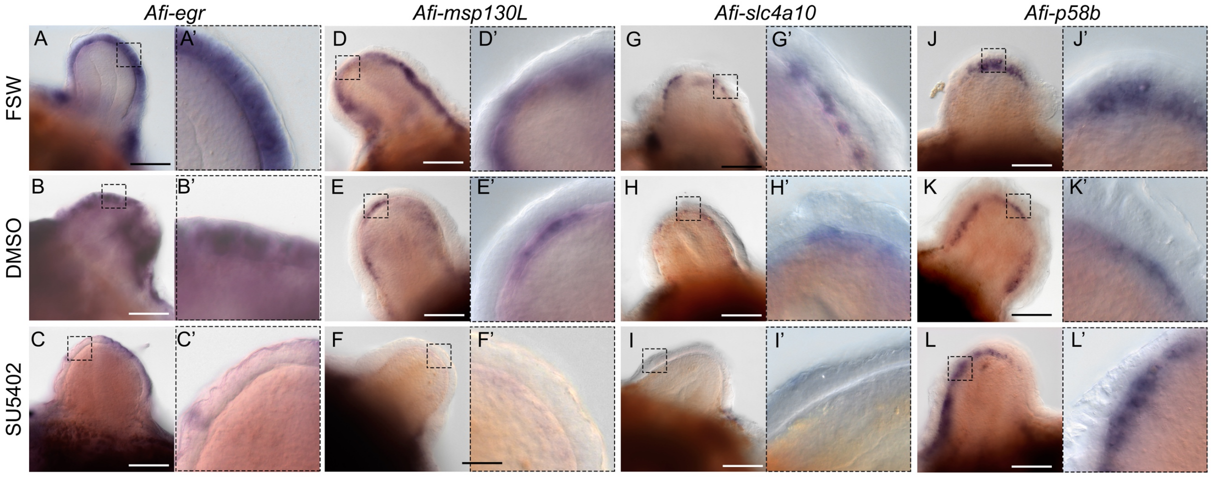

Figure S13: Spatial downregulation of selected genes in regenerating arm samples treated with SU5402 compared to controls. A-C) WMISH on regenerating arms shows normal expression of *Afi-egr* in epidermis is downregulated in SU5402-treated samples. A'-C') Same images at higher magnification. D-F) WMISH on regenerating arms shows normal expression of *Afi-msp130L* in the skeletogenic dermal layer is downregulated in SU5402-treated samples. D'-F') Same images at higher magnification. G-I) WMISH on regenerating arms shows normal expression of *Afi-slc4a10* in the skeletogenic dermal layer is downregulated in SU5402-treated samples. G'-I') Same images at higher magnification. J-L) WMISH on regenerating arms shows normal expression of control gene *Afi-p58b* in the skeletogenic dermal layer is maintained SU5402-treated samples. J'-L') Same images at higher magnification. Scale bars: 100μm.

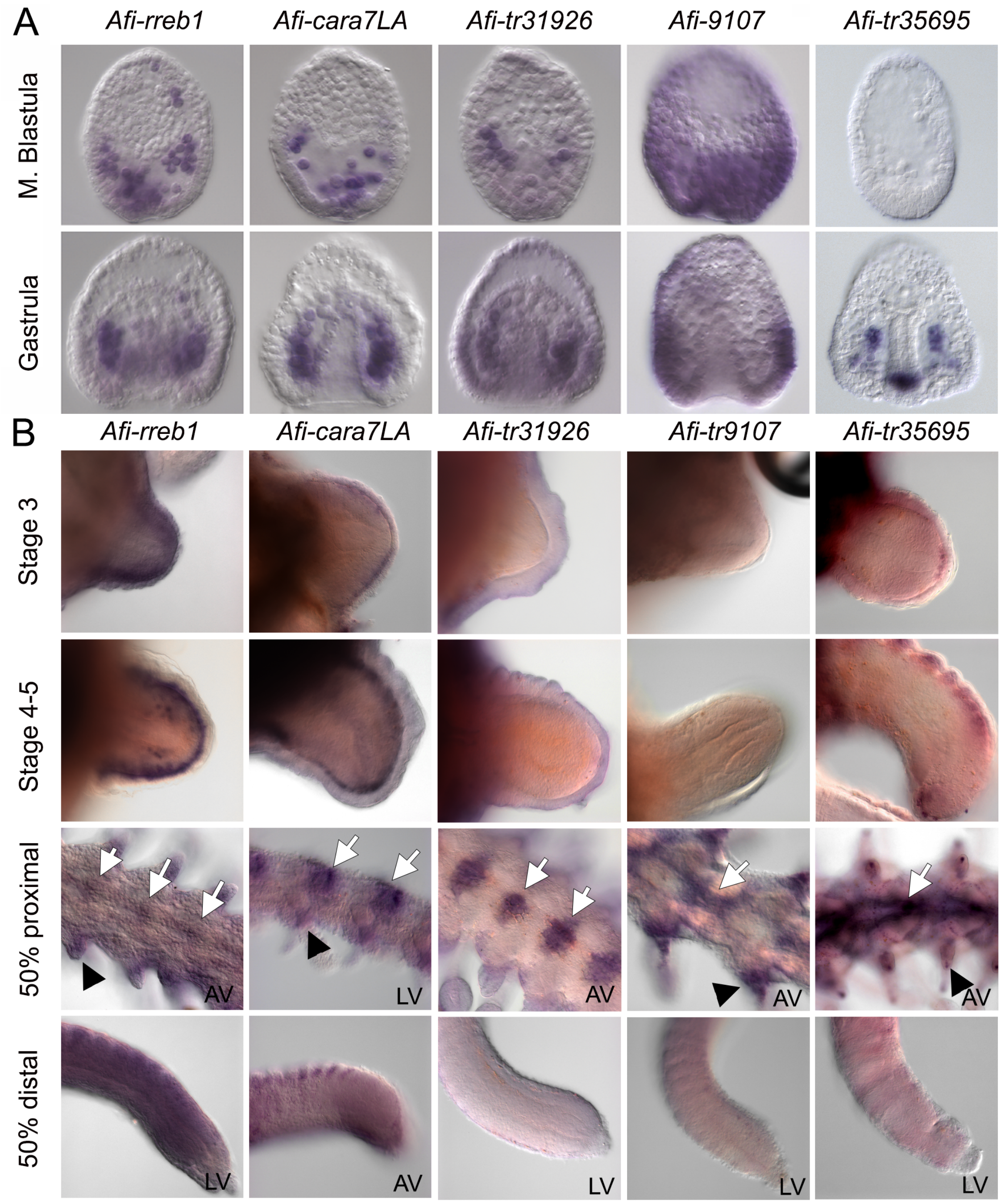

Figure S14: Novel identified genes are expressed in skeleton forming cells. WMISH on five genes: *Afi-rreb1, Afi-cara7La, Afi-tr31926, Afi-tr9107,* and *Afi-tr35695* at (A) mesenchyme blastula and gastrula stages of embryogenesis and (B) early and late stages of adult arm regeneration in the brittle star. All genes are expressed in skeletal cells sometime in development and regeneration. Exception is *Afi-tr9107* that shows expression in the ectoderm in a domain similar to the two ligands *fgf9/16/20* and *vegf3.* White arrows – expression in vertebrae, Black arrowheads – expression in spines. LV – lateral view, AV – aboral view. Embryos are all oriented with apical pole at the top and vegetal pole at the bottom; regenerating arms are oriented with proximal on the left and distal on the right.

Development • Supplementary information

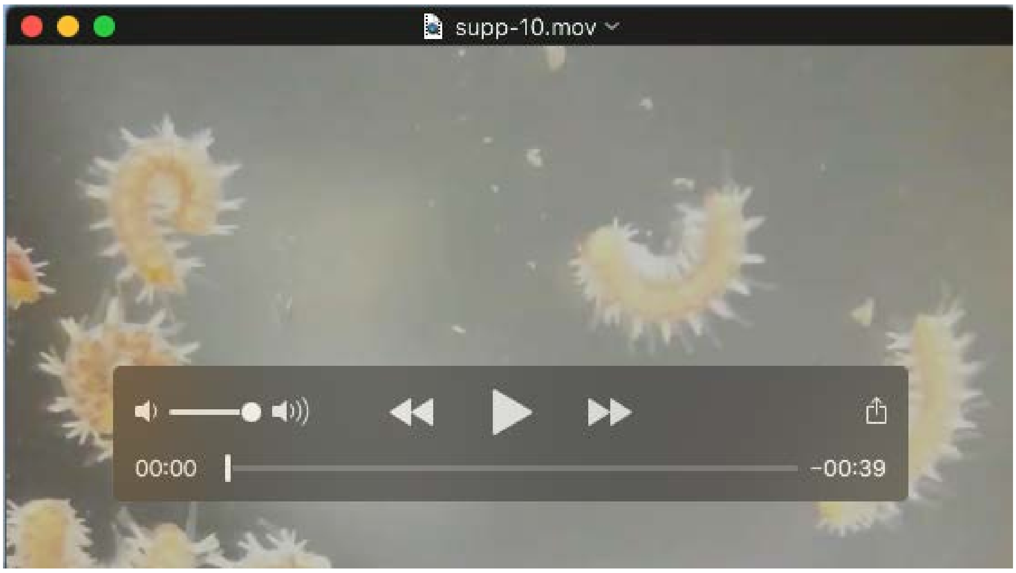

Movie 1: Control and SU5402-treated regenerating arm explants are alive and motile after 48h of treatment.

Table S1. Annotations of Fgf and Vegf genes using BLAST against Swissprot database.
eval, E value; pident, BLAST percentage identity.

Click here to download Table S1

Table S2. Heatmap of embryonic gene expression time-courses based on normalized Nanostring counts.
hpf, hours post fertilization.

Click here to download Table S2

Table S3. Heatmap of regenerating arm gene expression time-courses based on normalized Nanostring counts.
hpa, hours post amputation; NR, non-regenerating; St, stage; prox, proximal; dist– distal.

Click here to download Table S3

Table S4. Nanostring codeset sequences and their source.

Click here to download Table S4

Table S5. Summary of Nanostring experiments in embryos and regenerating adult arms of *A. filiformis* treated with SU5402.
Values shown are Log2(SU5402/DMSO). Threshold of significance is ±1. Positive values indicate upregulation, negative values indicate downregulation. TB, transcriptome batch; NB, Nanostring batch; N/D, not detected; Yellow highlight, internal standards. Red, significantly upregulated; Green, significantly downregulated.

Click here to download Table S5

Table S6. Summary table of differential transcriptome analysis of Fgf inhibition experiment.
Normalised values and log2 folds of difference (fc) are shown for 140 transcripts downregulated by SU5402 at 27 hours of embryonic development. Peptides encoded by the identified transcripts are annotated using BLAST against the *S. purpuratus* (SPU) genome and developmental transcriptome, functional annotation categories and BLAST against the NCBI non-redundant database.

Click here to download Table S6

Table S7. Summary table of protein prediction of five novel genes downregulated by FGF perturbation with unknown function.
BLAST results reveal that two of the genes have no immediate similarity to any other species found in the NCBI non-redundant (NR) database, while the other three find poor similarity with genes in other organisms. All genes can be identified in another brittle star species (*O. brevispinum* or *O. spiculata*) with high confidence and often at least partial hits were found in other eleutherozoa (echinoid *E. tribuloides* or asteroid *A. muricatum*) but not crinoids. Four of the five genes are likely to encode for secreted proteins due to the signal peptide prediction using SignalP. The CDART tool only found conserved domains in two out of the five genes.

Click here to download Table S7

Development • Supplementary information

Supplementary references:

1. Nguyen LT, Schmidt HA, Von Haeseler A, Minh BQ. IQ-TREE: A fast and effective stochastic algorithm for estimating maximum-likelihood phylogenies. Mol Biol Evol. 2015;32(1):268–74.
2. Altenhoff AM, Levy J, Zarowiecki M, Vesztrocy AW, Dalquen DA, Müller S, et al. OMA standalone : orthology inference among public and custom genomes and transcriptomes. Genome Reseach. 2019;29:1–12.
3. Altenhoff AM, Glover NM, Train CM, Kaleb K, Warwick Vesztrocy A, Dylus D, et al. The OMA orthology database in 2018: Retrieving evolutionary relationships among all domains of life through richer web and programmatic interfaces. Nucleic Acids Res. 2018;46(D1):D477–85.
4. Dylus DV, Czarkwiani A, Stångberg J, Ortega-Martinez O, Dupont S, Oliveri P. Large-scale gene expression study in the ophiuroid Amphiura filiformis provides insights into evolution of gene regulatory networks. Evodevo. 2016;7(1).
5. Czarkwiani A, Ferrario C, Dylus D V., Sugni M, Oliveri P. Skeletal regeneration in the brittle star Amphiura filiformis. Front Zool. 2016;13:18.

Development • Supplementary information
